# Sequence-Specific Targeting of Dosage Compensation in *Drosophila* Favors an Active Chromatin Context

**DOI:** 10.1371/journal.pgen.1002646

**Published:** 2012-04-26

**Authors:** Artyom A. Alekseyenko, Joshua W. K. Ho, Shouyong Peng, Marnie Gelbart, Michael Y. Tolstorukov, Annette Plachetka, Peter V. Kharchenko, Youngsook L. Jung, Andrey A. Gorchakov, Erica Larschan, Tingting Gu, Aki Minoda, Nicole C. Riddle, Yuri B. Schwartz, Sarah C. R. Elgin, Gary H. Karpen, Vincenzo Pirrotta, Mitzi I. Kuroda, Peter J. Park

**Affiliations:** 1 Division of Genetics, Department of Medicine, Brigham and Women's Hospital, Harvard Medical School, Boston, Massachusetts, United States of America; 2 Department of Genetics, Harvard Medical School, Boston, Massachusetts, United States of America; 3 Center for Biomedical Informatics, Harvard Medical School, Boston, Massachusetts, United States of America; 4 Department of Molecular Biology, Cell Biology, and Biochemistry, Brown University, Providence, Rhode Island, United States of America; 5 Department of Biology, Washington University in St. Louis, St. Louis, Missouri, United States of America; 6 Department of Molecular and Cell Biology, University of California Berkeley, Berkeley, California, United States of America; 7 Department of Genome Dynamics, Lawrence Berkeley National Lab, Berkeley, California, United States of America; 8 Department of Molecular Biology, Umeå University, Umeå, Sweden; 9 Department of Molecular Biology and Biochemistry, Rutgers University, Piscataway, New Jersey, United States of America; University of Cambridge, United Kingdom

## Abstract

The *Drosophila* MSL complex mediates dosage compensation by increasing transcription of the single X chromosome in males approximately two-fold. This is accomplished through recognition of the X chromosome and subsequent acetylation of histone H4K16 on X-linked genes. Initial binding to the X is thought to occur at “entry sites” that contain a consensus sequence motif (“MSL recognition element” or MRE). However, this motif is only ∼2 fold enriched on X, and only a fraction of the motifs on X are initially targeted. Here we ask whether chromatin context could distinguish between utilized and non-utilized copies of the motif, by comparing their relative enrichment for histone modifications and chromosomal proteins mapped in the modENCODE project. Through a comparative analysis of the chromatin features in male S2 cells (which contain MSL complex) and female Kc cells (which lack the complex), we find that the presence of active chromatin modifications, together with an elevated local GC content in the surrounding sequences, has strong predictive value for functional MSL entry sites, independent of MSL binding. We tested these sites for function in Kc cells by RNAi knockdown of *Sxl*, resulting in induction of MSL complex. We show that ectopic MSL expression in Kc cells leads to H4K16 acetylation around these sites and a relative increase in X chromosome transcription. Collectively, our results support a model in which a pre-existing active chromatin environment, coincident with H3K36me3, contributes to MSL entry site selection. The consequences of MSL targeting of the male X chromosome include increase in nucleosome lability, enrichment for H4K16 acetylation and JIL-1 kinase, and depletion of linker histone H1 on active X-linked genes. Our analysis can serve as a model for identifying chromatin and local sequence features that may contribute to selection of functional protein binding sites in the genome.

## Author Summary

The genomes of complex organisms encompass hundreds of millions of base pairs of DNA, and regulatory molecules must distinguish specific targets within this vast landscape. In general, regulatory factors find target genes through sequence-specific interactions with the underlying DNA. However, sequence-specific factors typically bind only a fraction of the candidate genomic regions containing their specific target sequence motif. Here we identify potential roles for chromatin environment and flanking sequence composition in helping regulatory factors find their appropriate binding sites, using targeting of the *Drosophila* dosage compensation complex as a model. The initial stage of dosage compensation involves binding of the Male Specific Lethal (MSL) complex to a sequence motif called the MSL recognition element [1]. Using data from a large chromatin mapping effort (the modENCODE project), we successfully identify an active chromatin environment as predictive of selective MRE binding by the MSL complex. Our study provides a framework for using genome-wide datasets to analyze and predict functional protein–DNA binding site selection.

## Introduction

In *Drosophila*, Male Specific Lethal (MSL) complex binds to the single male X chromosome to increase transcription approximately two-fold, in order to equalize the output of both female X chromosomes [2]–[4]. We have proposed that MSL complex locates its target binding sites using a two-step mechanism [5]. First, the complex distinguishes X from autosomes by binding a subset of 200–300 sites on X known as “chromatin entry sites” (CES) [6]–[8] or “high affinity sites” (HAS) [9], [10]. Recognition of CES is a sequence-dependent step, as these sites share a GA-rich motif [8], [10] designated the “MSL recognition element”, or MRE, whose function has been demonstrated by site-directed mutagenesis [8]. In contrast, the second targeting step lacks a consensus sequence but is strongly linked to transcription [11]–[14], with the complex locating active genes on the same chromosome [15].

**Figure 1:**
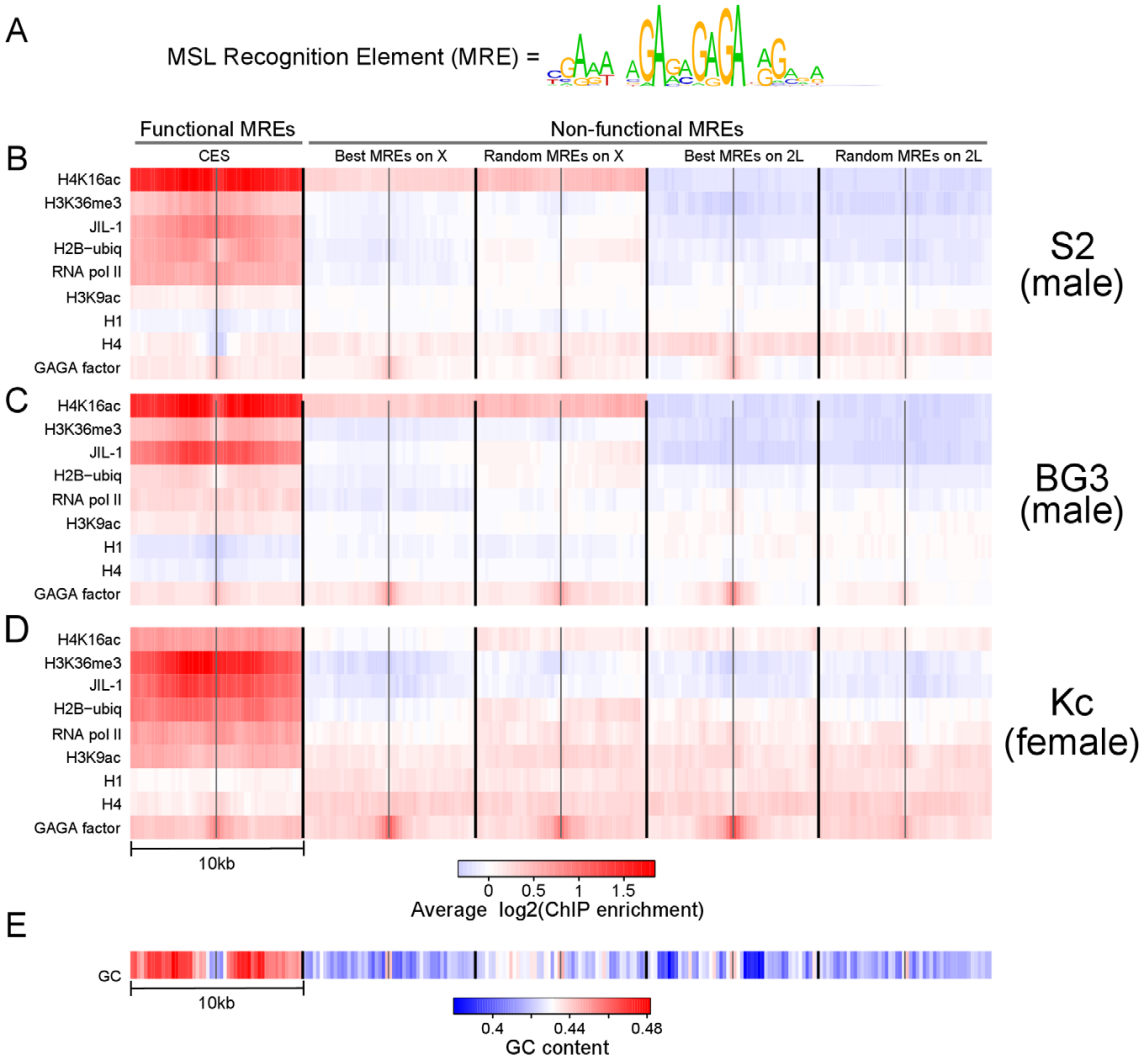
Active chromatin context and elevated GC content are associated with functional MSL recognition elements (MREs) on the X chromosome, independent of MSL binding. (A) The sequence motif for the MRE. (B–D) The average enrichment of various chromatin modifications or chromatin-binding proteins around (+/−5 kb) the functional and non-functional MREs is visualized using a heat map in each of three *Drosophila* cell lines: S2 in panel B; BG3 in panel C; and Kc in panel D. (E) A heat map showing the average GC content around each class of MREs. The data were obtained from genome-wide ChIP-chip profiles generated as part of the modENCODE project. Many active marks are broadly enriched around functional MREs in both male and female cell lines, suggesting that active chromatin is strongly associated with functional MREs independent of MSL binding. See also Figures S1 and S2.

CES were first identified in *msl3* mutant embryos, in which the initial, sequence-specific step of MSL binding occurs but the second, sequence-independent step does not [7], [8]. The MRE sequence motif was discovered based on the first 150 mapped CES (Figure 1A). CES function was tested in transgenes for the ability to attract MSL complex to autosomal insertion sites, and found to be dependent upon the intact MRE motif [8]. 150 is likely an underestimate of the total number of CES and functional MREs on X, as subsequent analysis of high occupancy MSL binding sites in wild type cells has revealed 309 peaks containing 379 MREs [8]. However, a conservative set of 150 should be sufficient to test for predictive features.

The MRE motif is only modestly enriched on the X chromosome compared to the autosomes. At a stringency where 137 of 150 CES contain the consensus motif (p-value of 10^−5^), there is a 1.8 fold higher MRE density on X compared to autosomes (on average 1 per 6 Kb on X, and 1 per 11 Kb on autosomes; Figure S1A), [8]. These average densities correspond to 12,481 total MREs in the genome, of which only 1 in 91 correspond to the set of CES considered here. Even if we restrict our attention to chromosome X, only 1 in 28 MREs maps to the CES set. Therefore, a key question is how functional MREs within CES are somehow recognized amongst a vast excess of un-utilized sites. That the MSL complex targets only a fraction of potential MRE sites for initial binding is a characteristic it shares with many sequence-specific binding factors whose predicted target motifs are often in vast excess to the sites actually utilized [16], [17]. Here, we investigate whether chromatin features influence binding site selection, using the MSL complex and a large compendium of genome-wide ChIP-chip profiles generated by the NHGRI modENCODE project as a model [18]. Our results support a model in which active chromatin composition and intrinsic GC content help define the initial binding sites of the MSL complex.

## Results

### An active chromatin context is predictive of functional MREs

To search for chromatin features that can distinguish functional MREs from those that do not recruit MSL complex (*i.e.*, non-functional MREs), we defined five classes of MREs in the *Drosophila* genome. The first set consists of 137 MREs that were experimentally defined by MSL complex binding [8], as discussed above. We called this set “Functional MREs”. The remaining four sets of 150 sequences each consist of the MREs that have the best consensus motif matches on either X or a control autosomal arm (2L) (“Best on X” and “Best on 2L” respectively), and 150 MREs chosen at random from either X or 2L (“Random X” and “Random 2L” respectively). We note that, in general, functional MREs display a broad range of motif binding specificity rather than being the best matches to the MRE consensus motif (Figure S1B). The result of this analysis is not affected by the choice of chromosome arm or the choice of random MREs (data not shown).

In each of the five classes of MREs, their locations are distributed along the length of the chromosome arm with no obvious clustering (Figure S1C). Within each set of sequences, we calculated the average profiles of various chromatin marks mapped by the modENCODE Drosophila Chromatin Consortium using genome-wide ChIP-chip. The average ChIP enrichment profiles for 10 kb regions centered around the motif in two male cell lines (S2 and BG3) are shown in Figure 1B–1C. We found that a number of chromatin marks associated with active transcription are strikingly enriched near functional MREs in CES, and not in the best or random MRE classes on X or autosomes. These include RNA pol II, H3K36me3, H3K9ac, and H2B-ubiquitin. In addition, functional sites are relatively depleted for core histone H4 and linker histone H1. Consistent binding of the sequence-dependent GAGA factor [19] across categories serves as an important control to demonstrate that GA-rich elements are broadly represented in each group of MREs. Another notable feature of these profiles is the enhancement of H4K16ac on the X chromosome as a whole [20]–[22], with additional enrichment of H4K16ac on true CES (Figure 1B–1C). Since enrichment of H4K16 acetylation is a known consequence of MSL targeting, we proceeded to ask whether the observed difference in chromatin context for other chromatin marks at CES might simply be a consequence of MSL binding rather than a contributing factor.

### Predictive chromatin marks are independent of MSL binding

To test whether the observed difference in chromatin context at CES is a consequence of MSL binding rather than a contributing factor, we examined the profiles of the same subset of chromatin marks in female Kc cells. Interestingly, we found that the set of marks that correlates with MRE function in male S2 cells were likewise informative in female Kc cells, in the absence of MSL complex (Figure 1D). The enrichment of H4K16 acetylation on the male X is notably less pronounced in female Kc cells lacking MSL complex. Still, this mark is enriched over functional MREs, consistent with previous observations of MSL-independent H4K16 acetylation at active genes on all chromosomes in males and females [21], [22]. Most strikingly, H3K36me3 and JIL-1, are enriched in functional MREs in both male and female cells, suggesting that these marks are independent of MSL binding at CES.

We also examined MREs for intrinsic sequence composition to search for correlations with function (Figure 1E). Surprisingly, we found a marked elevation of GC content in the 10 Kb flanking region surrounding functional MREs, coupled with a decrease in GC content in the 1 Kb nearest the functional MREs. Taken together, our results support a model in which local sequence characteristics and the active chromatin context of functional MREs may facilitate their initial selection.

**Figure 2:**
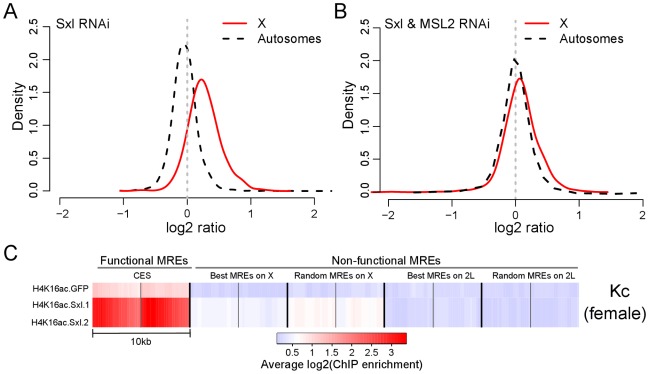
Ectopic upregulation of MSL2 by *Sxl* RNAi treatment induces dosage compensation of X-linked genes in female Kc cells by preferentially targeting MREs in an active chromatin context. (A) Distribution of gene expression ratios after *Sxl* knockdown in X chromosome and autosomes compared to control. The *y*-axis (density) represents the scaled proportion of the number of genes for a given log2 expression ratio (*x*-axis). Repression of *Sxl* leads to ectopic expression of MSL2, which results in dosage compensation of the X chromosome. (B) Distribution of gene expression ratios after *Sxl* and MSL2 double knockdown for X chromosome and autosomes compared to control. In the absence of functional MSL2, no dosage compensation is observed. (C) A heat map showing the average enrichment of H4K16 acetylation (H4K16ac) around (+/−5 kb) the functional and non-functional MREs in control (GFP) and after two independent *Sxl* RNAi knockdowns. The gene expression data in panels A and B are based on the same cells as replicate 1 in the heat map.

Next, we wanted to directly test the potential of MREs in an active chromatin environment in females to recruit MSL complex. The key female sex determination protein, SXL, represses dosage compensation by inhibiting MSL2 translation [23], [24]. Loss of SXL results in the expression, stabilization, and targeting of the MSL complex in female cells [25]. Therefore, we depleted SXL by RNA interference (RNAi) in Kc cells [26]. Upon treatment, we observed a general, MSL2-dependent increase in transcription from the female X chromosomes, consistent with a partial induction of dosage compensation (Figure 2A–2B). The increase in X-linked gene expression did not reach the maximum theoretical amount for perfect compensation (log_2_2 = 1), consistent with observations that MSL-independent ploidy effects can also contribute to overall compensation [27], [28].

The induction of MSL complex in Kc cells allowed us to ask whether ectopic MSL complex in female cells recognized the same functional subset of MREs on X as in male cells, by examining the distribution of H4K16 acetylation as a mark of MSL function. We found that Sxl RNAi induces high levels of this modification preferentially at the same MREs that are functional in males (Figure 2C), supporting the idea that MSL complex recognizes MREs in an active chromatin context. Our findings with MSL complex parallel recent results for the heat shock transcription factor HSF [29], suggesting that sequence-specific DNA binding factors may generally utilize chromatin context to facilitate selective targeting within a complex eukaryotic genome.

### Identification of the best chromatin marks for prediction of functional MREs

Since specific chromatin marks are enriched near MREs that are utilized compared with those that are not, we next asked whether these marks provide enough information, either individually or in combination, to explain the MSL entry site binding pattern on X. To address this question, we systematically investigated the predictive power of the chromatin marks for functional MREs in Kc cells, which have the potential for MSL targeting but do not express the MSL complex. We asked whether we could build a simple prediction model based on individual or a combinations of chromatin features in Kc cells that would distinguish functional MREs from non-functional ones in male S2 and BG3 cells, where MSL complex is expressed.

**Figure 3:**
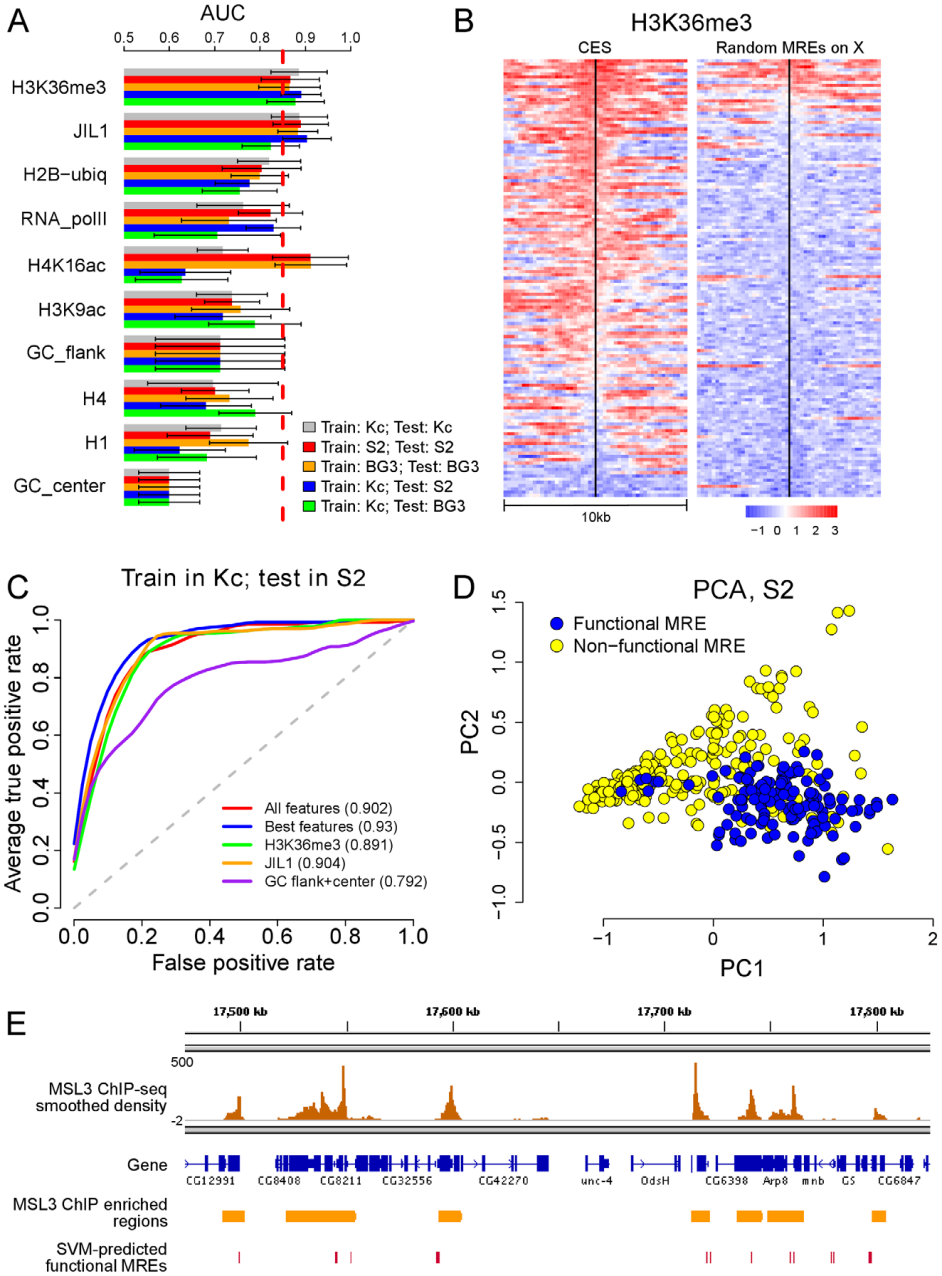
Chromatin context is predictive of functional MREs. (A) A bar plot showing the performance of individual chromatin features for distinguishing functional and non-functional MREs using various training and testing schemes. The best features are H3K36me3 and JIL1. H4K16ac shows strong association with the presence of functional MREs in both male cell lines but much weaker association in female Kc cells, supporting the known role of H4K16ac as a key consequence of MSL binding. (B) A heat map showing the distribution of the H3K36me3 mark around individual functional and non-functional MREs on the X chromosome in female Kc cells, ordered by the level of enrichment at the MREs. (C) Sensitivity (*y*-axis) and 1 - specificity (*x*-axis) of prediction using a support vector machine (SVM) are shown using a receiver operator characteristic (ROC) curve. This indicates that chromatin features in female Kc cells can be used to predict the identity of functional MREs in male S2 cells with high sensitivity (high true positive rate) and specificity (low false positive rate). (D) A principal component projection of the functional and non-functional MREs based on the best chromatin features in S2 cells, showing that the two MREs groups can be well-separated. See also Figure S3. (E) A genome browser view that shows the overlap of SVM-predicted functional MREs and MSL binding sites identified by an independent MSL3 ChIP-seq analysis.

We first tested whether individual chromatin features could discriminate functional MREs from non-functional ones. A chromatin feature is defined by its average ChIP enrichment within the 10 kb region surrounding each MRE. In addition, we defined two features to represent the average GC content near the MRE (center 1 kb) or in its flanking regions (10 kb excluding the center 1 kb). To test whether individual or combinations of features could distinguish the functional MREs from the non-functional ones, we used support vector machine (SVM), a classification algorithm demonstrated to have excellent performance in a wide range of problems [30], [31]. Briefly, the set of ChIP enrichment at each MRE is treated as a feature vector of that MRE. Given a set of training samples, SVM calculates an optimal hyperplane that can separate non-functional MREs from functional MREs in the feature space. Here we used a SVM with a radial basis kernel that is implemented in the R package e1071 (See Methods and Materials). To accurately estimate predictive power, and to avoid the potential bias due to using the same set of CES and non-functional MRE genomic locations in S2, BG3 and Kc cell lines, we evaluated the predictive power of a feature using 10-fold cross-validation. In this scheme, we withhold a random 10% of the MREs, build a model based on the remaining 90%, measure how well the model predicted the functionality of the withheld MREs, and repeat this process multiple times to obtain the average performance. The cross-validation result is presented using a standard measure called the Area Under Curve (AUC) of Receiver Operating Characteristic [32] curve. The ROC curve (examples are shown in Figure 3C) quantifies the sensitivity and specificity of classification by estimating true and false positive rates over all threshold values, and the AUC summarizes the curve with a single number. A random predictor receives an AUC of 0.5, and a perfect predictor achieves an AUC of 1 [32].

By comparing the AUC of each individual chromatin feature in Kc, S2 and BG3 cells, we observed that many active chromatin marks could distinguish functional from non-functional MREs (Figure 3A). Among all the features tested, H3K36me3 was the best predictor in all three cell lines (mean AUC of 0.884), followed by JIL-1 (mean AUC of 0.864). Interestingly, we had previously speculated that H3K36me3 might be involved, based on MSL affinity for this mark in active genes [8], [12], [14]. Since H3K36me3 and JIL-1 are enriched in Kc cells at predicted CES even without MSL binding, they could prime functional MREs for sequence-specific MSL binding, or be coincident with true causative factors. Nearly all putative CES in Kc cells are embedded in a chromatin environment enriched for H3K36me3 (Figure 3B), however, we previously determined that when H3K36me3 is depleted, there are still enough features for the MSL complex to distinguish MREs [12]. Since no single feature may be sufficient to drive MSL recognition, we next asked whether combinations of marks and local sequence composition might further improve predictive power for CES.

### Predictions utilizing combinations of chromatin marks and local GC content

On average, the GC content of CES is similar to the random or best MREs in the 1 Kb of sequence immediately surrounding the motif, but the GC content consistently rises in flanking sequences, to produce a distinctive average profile (Figure 1E). Examination of the individual heatmaps confirms that this is a broadly consistent characteristic of CES (Figure S2). We found that the relative GC content in *D. melanogaster* is elevated in genes compared to intergenic sequence (44% vs. 41%) [33] so it is not surprising to see this characteristic in conjunction with the active gene clusters where CES are found. However, the distinctive shape of the profile, with low GC immediately surrounding the CES, is unexpected and not seen with autosomal MREs, even when associated with H3K36me3 and thus presumably analogous active gene clusters (Figure S2). The significance of this intrinsic feature clearly merits future experimental analysis. However, GC profile alone does not appear to provide enough information to predict functional MREs with high accuracy (Figure 3C).

To search for combinatorial marks that might distinguish functional MREs, we compared the predictive power of the SVM generated using every possible combination of features in our dataset (Figure S3A–S3B). We found that the best individual features (H3K36me3 and JIL-1) performed very well, similarly to the best combinations of features when SVM trained using Kc data, and tested on S2 or BG3 data (Figure 3C). There are many combinations of features that predict functional MREs with high accuracy. In general, the best performing combinations included the following: (1) H3K36me3 or JIL-1; (2) H2B-ubiq or H3K9ac; (3) a core histone; and (4) GC content (Figure S3A–S3B). The excellent performance of this combination is consistent with the identification of these factors as core features by feature correlation analysis (Figure S3C).

The cross-validation results are summarized in the ROC plots in Figure 3C and Figure S3D. Although not perfect, the best combination separates the functional and non-functional MREs with high accuracy (mean AUC = 0.931), as visualized in principal component space in Figure 3D. Each MRE can be considered as a point in multi-dimensions, with each axis as the enrichment level for a mark; to show the data in two-dimensions, we define new axes, called principal components, which are combinations of the original variables satisfying certain desirable properties. We can indeed observe a good separation of the functional from non-functional MREs in this view (Figure 3D).

### Genome-wide prediction of functional MREs

The analyses presented so far focused on only a subset of clearly functional and non-functional MREs. This allowed us to effectively identify chromatin features that can distinguish functional MREs from non-functional ones, and to build a predictive SVM model for functional MREs. Here we extend our analysis to test whether our model could accurately select additional functional MREs genome-wide. We trained an SVM model with Kc cell chromatin features and then tested its ability to eliminate non-functional MREs on autosomes and chromosome X using S2 data. The SVM algorithm selects the decision threshold that optimally separates functional from non-functional MREs, as confirmed in Figure S3E, and the overall AUC of this prediction is about 0.84 (Figure S3F). We specifically tested different individual and combinations of features. Using the best combination of features, our SVM model can eliminate over 75% of candidate MRE sites on X, and ∼85% of candidate sites on autosomes, while retaining almost all of the functional MREs on the X chromosome (up to 94%) (Table S2). Almost 10,000 non-functional sites are eliminated using our model, with retention of approximately 1600 MREs genome-wide. Approximately half (763) of those remaining MREs map to the X chromosome but are not included in the conservative set of 137 CES.

We suspect that the large number of remaining MREs on the X chromosome indicates that there may be more true MSL binding sites than the set of 137 CES we used in this study. Therefore, we asked how many of these additional 763 MREs overlap with previously mapped MSL binding sites identified by MSL3 ChIP-seq [8]. Of the 763 SVM-predicted functional MREs on X, 503 overlapped with an MSL binding site (Figure 3E). This suggests that the actual false positive rate on the X chromosome may be as low as 260/3343 = 7.8%. This is slightly lower than the false positive rates for the autosomes (895/8144 = 11%) (Table S2), likely due to the fact that even though most strong peaks identified in the MSL3 ChIP-seq data with stringent criteria are CES [8], some may be sites to which MSL complex spreads.

### Increased lability of nucleosomes at chromatin entry sites correlates with MSL binding

**Figure 4:**
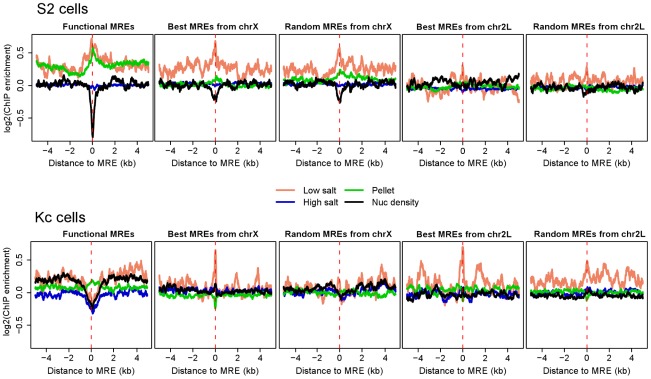
Increased lability of nucleosomes at chromatin entry sites correlates with MSL binding. Average enrichment profiles of chromatin properties around MREs are shown for S2 and Kc cells. Nucleosome density and successive salt extracted fractions of MNase-treated chromatin from S2 and Kc cells were described previously [28]. Profiled chromatin properties comprise nucleosome density (black) and successive salt extracted fractions of MNase-treated chromatin: low salt (pink, 80 mM NaCl), high salt (blue, 600 mM NaCl), and pellet (green, salt insoluble). The dashed red lines on each plot indicate the MRE centers. In male S2 cells, nucleosomes are more labile at the MREs on the X chromosome in general, and nucleosome density is especially low at functional MREs. The relative lability of nucleosomes on the X chromosome is not seen in female Kc cells, and the extent of nucleosome depletion at functional MREs is less pronounced. See also Figure S4.

In addition to enrichment for marks associated with active genes, we also saw relative depletion of histone H1 over functional MREs (Figure 1), and, from previous work, depletion of H3 and thus presumably nucleosomes themselves [8], [10]. Therefore, we examined modENCODE data from S2 and Kc cells on the release of nucleosomes following salt extraction to determine whether functional MREs are packaged into more labile chromatin when compared to non-utilized MREs [34]. We found that in male S2 cells functional MREs are depleted for histones in general, and enriched for nucleosomes that are extracted in low salt or remain in the pellet after high salt extraction (Figure 4), both fractions that were previously characterized as enriched in regulatory regions [35]. In addition, the two sets of non-functional MREs on the X chromosome appeared to have a milder, but discernable increase in nucleosome lability when compared to the MREs on autosomes, consistent with the observation that X chromosome in male S2 cells generally adopts a more open chromatin conformation [36].

In contrast, CES MREs in female Kc cells exhibit a modest average decrease in nucleosomal occupancy (Figure 4), but intriguingly, this appears to be a difference at a subset of sites rather than the entire set (Figure S4). Notably, the entire X chromosome in Kc cells does not appear to be packaged in a more open chromatin state compared to autosomes (Figure 4). We conclude that strong nucleosome depletion and a more open chromatin conformation on X are mainly consequences rather than causes of MSL binding.

### JIL-1 kinase enrichment and histone H1 depletion on active gene bodies of the MSL–bound X chromosome

**Figure 5:**
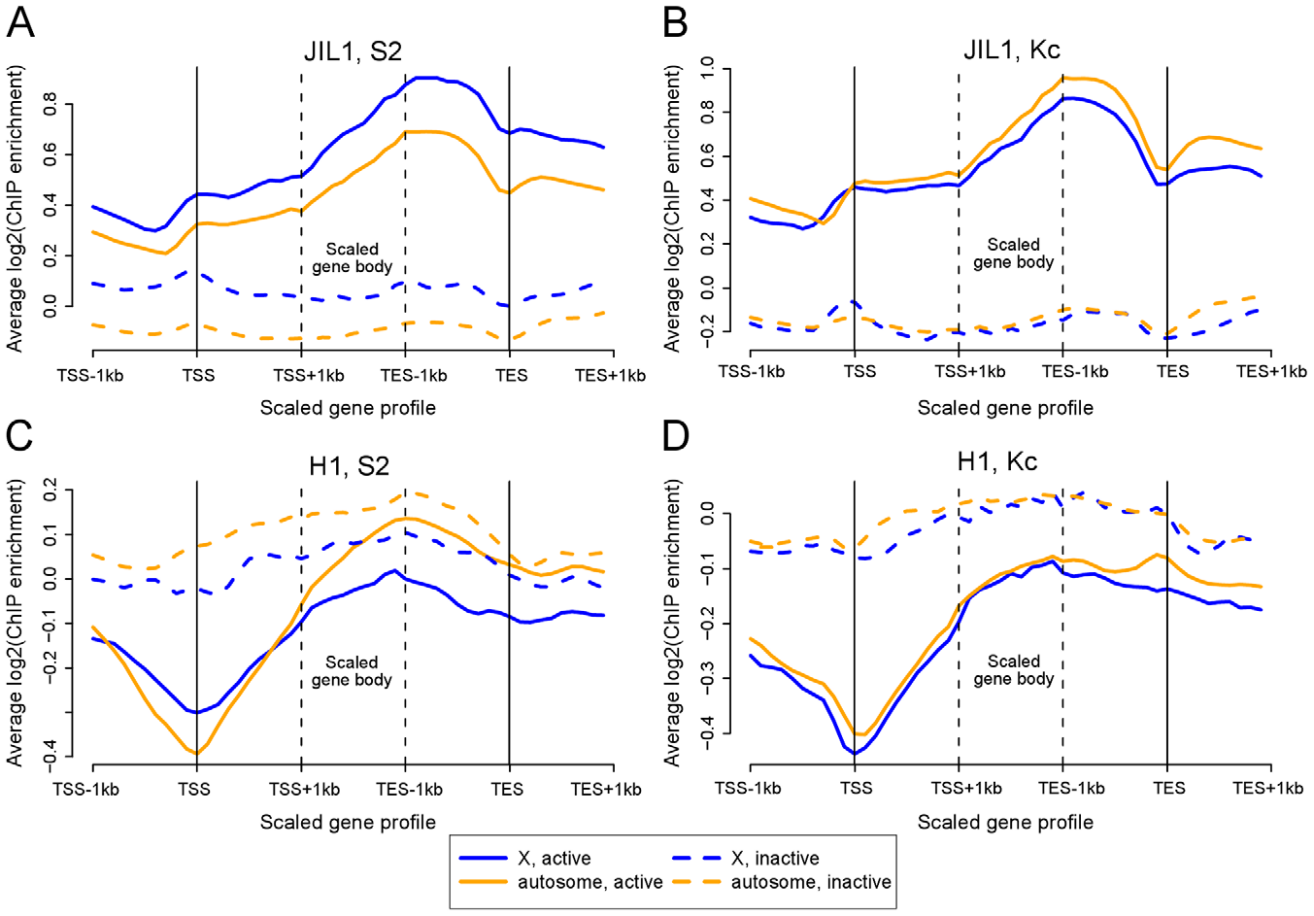
The bodies of active X-linked genes are enriched for JIL-1 kinase and depleted for histone H1 in S2 but not Kc cell lines. Each panel shows the average scaled ChIP enrichment profile (meta-gene profile) of active and inactive genes located on X chromosome and autosomes. (A) JIL-1 in S2 cells, (B) JIL-1 in Kc cells, (C) H1 in S2 cells, and (D) H1 in Kc cells. Notably, JIL-1 is enriched along the gene bodies of X-linked genes in males, while histone H1 is depleted. See also Figure S5.

Once MSL complex identifies the male X, we have proposed that it spreads to affect the active genes on the chromosome as a whole [5]. In addition to the core MSL complex consisting of proteins that are essential in males but not females (MSL1, MSL2, MSL3, MOF, and MLE), the JIL-1 kinase is known to be enriched on the male X [37]. JIL-1 is essential in both males and females and binds interband regions on all chromosomes in both sexes [38]. Since we observed that JIL-1 is enriched at functional MREs (Figure 1 and Figure 3A), we tested whether it is also associated with active gene bodies. We constructed average scaled profiles of JIL-1 binding (meta-gene profiles) of all genes greater than 2 kb in length based on the FlyBase dm3 gene annotation [39]. Gene expression in the three cell lines was determined by RNA-seq data (Figure S5) [34]. We found that JIL-1 binds active gene bodies, with a bias towards 3′ ends, on all chromosome arms (Figure 5A). On the male X, this pattern is increased in its intensity above the level on autosomes and correlates strongly with binding of the MSL complex since the increased occupancy is not seen on female X chromosomes (Figure 5B). These results are consistent with previous polytene immunostaining [37] and ChIP analyses [40].

In contrast to JIL-1, the linker histone H1 shows depletion on active genes (Figure 5C). Interestingly, in male cells this depletion is more prominent on X-linked gene bodies than on autosomal gene bodies, whereas X and autosomes show no obvious difference in female cells (Figure 5D), consistent with previous polytene immunostaining results [41]. These two results further underscore the distinct character of the *Drosophila* male X chromosome once MSL complex is bound to active genes.

When examining our modENCODE data, we noticed that many additional data sets are slightly enriched on the entire X chromosome compared to autosomes in male S2 cells, but not in female Kc cells (Figure S6, and Table S3). X-chromosome-wide enrichment of nucleoporins, Megator and Nup153 have also been observed in male S2 cells, but not in female Kc cells [42]. These results are unlikely to simply reflect increased access of antibodies to X chromatin in the ChIP procedure, because histone H1 shows the opposite trend. Therefore, these enrichments may be related to dosage compensation of the male X, either directly, as is the case for H4K16ac, JIL-1, and possibly RNA pol II, or indirectly, as a consequence of the more open chromatin environment created by the dosage compensation mechanism.

### MSL1 and H4K16 acetylation are found on virtually all active X linked genes in male cells

**Figure 6:**
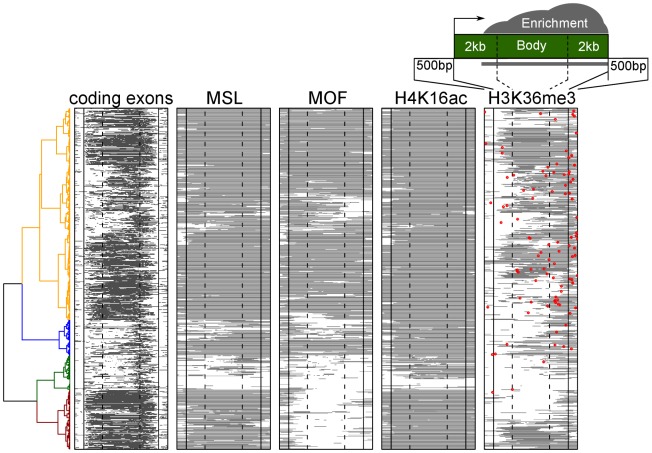
MSL1 and H4K16 acetylation are found on virtually all active X linked genes in male S2 cells. The plot shows the positions of exons, and the regions of enrichment for MSL1, MOF acetyltransferase, H4K16ac, and H3K36me3 along the bodies of active X linked genes. Each row represents an active gene scaled to the same size. The genes were clustered based on the chromatin features. These profiles show that CES (red dot on the H3K36me3 map) are located closer to the 3′ end in general and are embedded within domains enriched for H3K36me3 as well as MSL1, MOF, and H4K16ac.

MSL-dependent dosage compensation is thought to be an organism-wide phenomenon in *Drosophila* males, excluded from the germline [43], but otherwise not restricted to particular tissues or developmental time points. Classical mitotic recombination experiments support a requirement for MSL proteins throughout development in dividing tissues [44]. In addition, comparison of gene expression in males and females demonstrates that the vast majority of X-linked genes are up-regulated in males [45]. However, stable MSL binding appears to be more restricted than its functional consequence, H4K16 acetylation [22], so we wondered whether binding favored particular types of genes. To examine this, we plotted the chromatin marks and MSL binding along active X linked genes in S2 cells (>2 Kb long), asking if clustering might define genes of particular structure, expression level, or gene ontogeny category (Figure 6). We found that MSL1 and H4K16 acetylation cover the vast majority (86%) of active X linked genes. Apparent exceptions (green cluster) are interesting as those genes also lack H3K36me3 (Figure 6) and H2B-ubiquitin (data not shown), two prominent marks of transcription [18].

While MSL1 and H4K16ac are associated with the bodies of virtually all active X linked genes in male S2 cells, the MOF H4K16 histone acetyltransferase was notably absent from a subset (dark brown cluster at bottom of Figure 6). Most of these genes still showed limited MOF enrichment around the promoter region, but not within the gene bodies as is characteristic of MSL complex. We were unable to identify any feature, such as relative expression level or chromatin state, that would distinguish these genes from the genes that showed MOF enrichment.

Since functional MSL chromatin entry sites are also preferentially associated with active gene marks, we asked where CES map relative to the set of clustered genes on X, indicating the location of CES by red dots on the gene structures in Figure 6 (H3K36me3 column). Our results demonstrate that CES are located at variable positions relative to active genes, with a bias towards their 3′-ends. Interestingly, the subset of genes lacking strong MOF binding within gene bodies also lack nearby entry sites, in agreement with the observation that MSL binding is strongest in the close vicinity of mapped chromatin entry sites [10], [46].

## Discussion

Genetic, genomic, and biochemical analyses in eukaryotes have revealed that DNA binding motifs alone are insufficient to explain the selective occupancy or specificity of regulatory factor function [16], [17]. The number of predicted binding sites is often vastly greater than the number of sites actually utilized. Therefore, a very important question in transcriptional regulation is how to identify additional parameters that must govern accurate binding site selection.

In this study, we considered the roles of chromatin environment and flanking sequence composition in selection of functional binding sites by a sequence-specific protein complex. It is generally not clear whether the chromatin features that are often observed at the binding sites of proteins contribute directly to binding selectivity or are simply a consequence of binding. In the dosage compensation system of the X chromosome in *Drosophila*, we had a unique opportunity to address this question because we can compare the chromatin environment of MSL binding sites in female cells, in the absence of the complex, to male cells, where the functional sites are bound. We also utilized binding data from an RNAi experiment in which we knocked down a component of the sex determination pathway in females to induce dosage compensation. Our bioinformatic analysis of a large number of profiles from the modENCODE project suggests that a pre-existing active chromatin context plays a critical role in establishing the initial binding of the MSL complex on the X. We also made the surprising discovery that GC content in the DNA surrounding functional binding sites has a characteristic profile.

**Figure 7:**
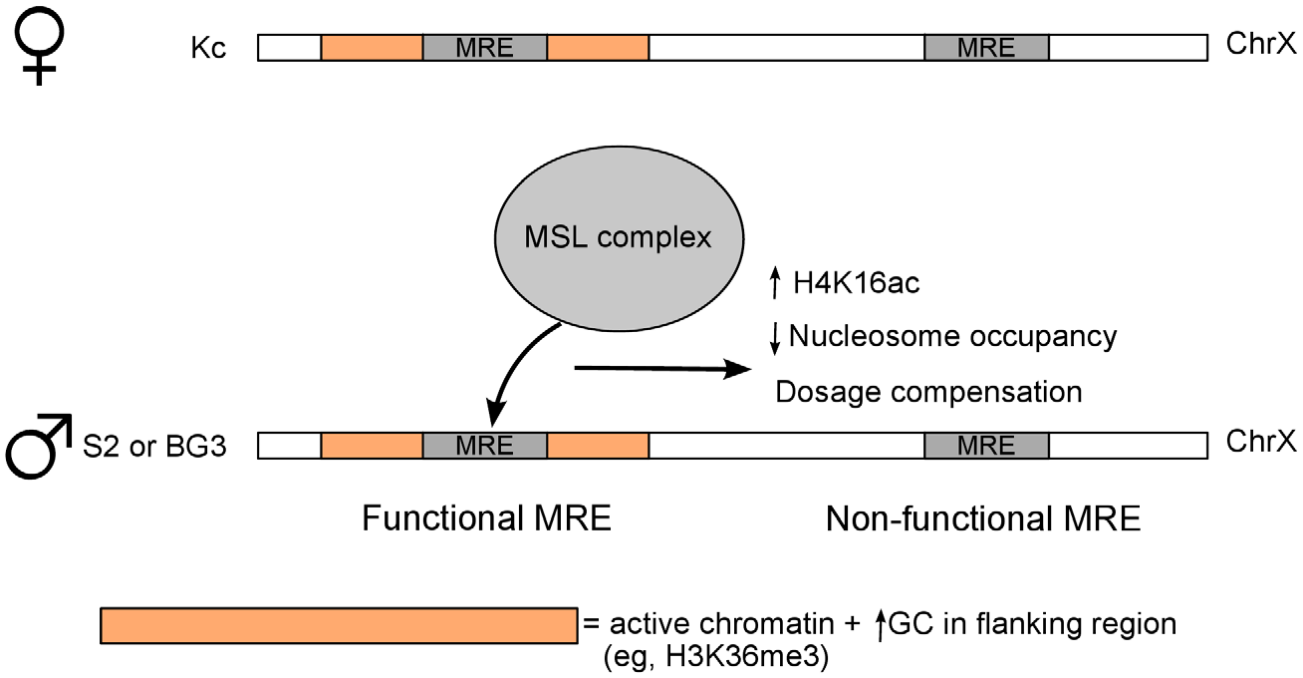
Model for binding site selection by a chromatin associated factor. Our results support roles for local chromatin environment and flanking GC content in discrimination of true target sites of the MSL dosage compensation complex. The model depicts the GC content and active chromatin marks surrounding MREs in female Kc cells that predict binding by MSL complex in male S2 or BG3 cells (or after MSL induction in female Kc cells). MREs that do not pre-exist in a favorable environment are not bound by MSL complex and thus are non-functional. Definition of the favorable chromatin features that pre-exist factor binding may be a general tool, in addition to DNA motif analysis, for prediction of functional binding sites.

In summary, our results strongly support a model in which an active chromatin composition helps define the initial entry sites selected by the MSL complex (Figure 7). Functional MSL binding results in increased lability of local nucleosomal composition, and H4K16 acetylation and JIL-1 binding along the bodies of virtually all active X-linked genes. Our work provides key insights into the order of events leading to dosage compensation in *Drosophila*, and can also serve as a model for using genome-wide data sets to understand how sequence-specific factors find their ultimate targets.

## Materials and Methods

### modENCODE ChIP-chip data processing

The majority of the ChIP-chip data are from the modENOCODE project [18]. Genomic DNA Tiling Arrays v2.0 (Affymetrix) were used to hybridize both ChIP and input DNA. We obtained the log-intensity ratio values (M-values) for all perfect match (PM) probes: M = log2(ChIP intensity)−log2(input intensity), and performed a whole-genome baseline shift so that the mean of M in each microarray is equal to 0. The smoothed log intensity ratios were calculated using LOWESS with a smoothing span corresponding to 500 bp, combining normalized data from two replicate experiments. All data are publicly accessible online through the modENCODE project (URL listed in Table S1). Data analysis was performed in R statistical programming environment (http://www.r-project.org). For the visualization of the heatmap (e.g., Figure 1), the +/−5 kb region surrounding each MRE was separated into non-overlapping bins of 200 bp. The smoothed probe value within each bin is averaged to obtain the enrichment value for that bin.

### Local sequence GC content

The GC content around each nucleotide is defined as the proportion of G or C in the closest 101 bp (ie, the target nucleotide, 50 bp upstream and 50 bp downstream). Similar to the ChIP-chip data, we separated the +/−5 kb region surrounding each MRE into non-overlapping bins of 200 bp. The average GC content in each bin represents the average of the GC content of the 200 bp within that bin.

### RNAi followed by expression analysis and ChIP–chip

We generated double-stranded RNA (dsRNA) to target GFP (negative control) or *Sxl* transcripts (amplicons designed by the *Drosophila* RNAi Screening Center (www.flyrnai.org), as described previously [22]. The following primer sets were utilized to amplify PCR products to template dsRNA synthesis:

GFP

F 5′-TAATACGACTCACTATAGGGAGAGGTGAGCAAGGGCGAGGAGCT-3′

R 5′-TAATACGACTCACTATAGGGAGATCTTGAAGTTCACCTTGATGCCG-3′

*Sxl* (DRSC21490)

F 5′-TAATACGACTCACTATAGGGAGAGATCACAGCCGCTGTCC-3′

R 5′-TAATACGACTCACTATAGGGAGATACCGAATTAAGAGCAAATAATAA-3′

*Sxl* (DRSC28896)

F 5′-TAATACGACTCACTATAGGGAGACCCTATTCAGAGCCATTGGA-3′

R 5′-TAATACGACTCACTATAGGGAGAGTTATGGTACGCGGCAGATT-3′

For expression analyses, GFP and Sxl DRSC21490 RNAi was performed in Kc cells using 6-well plates as described [47].

For ChIP-chip, RNAi using GFP, Sxl DRSC 21490, and Sxl DRSC28896 amplicons in Kc cells was scaled up to T225 flasks and chromatin preparation, and H4K16ac ChIP was performed using anti-H4K16ac antibody (Millipore, 07-329) and custom Nimblegen tiling arrays as described [22].

Kc GFP H4K16ac ChIP-chip datasets were published previously [22]: Gene Expression Omnibus accession numbers: GSM372470 (replicate #1) and GSM372471 (replicate #2).

### Cross-validation

We used 10-fold cross-validation to estimate the predictive power of a classification model based on a training dataset (e.g., chromatin feature in Kc cells) and a test dataset (e.g., chromatin feature in S2 cells). Each sample in a dataset is an MRE, a feature is a histone modification (e.g., H3K36me3) or a chromatin binding protein (RNA Pol II), and the label for each sample is either “Functional MRE” (positive class) or “Non-functional MRE” (negative class). The aim is to train a prediction model that can distinguish functional from non-functional MREs based on the chromatin features. In a 10-fold cross-validation, the training data are randomly divided into 10 equal-sized portions in which the same proportion of positive and negative samples are preserved in each portion. In each of the 10 iterations, the data from nine portions are used to train a predictive model, while the remaining one portion is used to test the performance of the prediction model. Performance is measured by true positive rate (sensitivity) and false positive rate (1-specificity). The tradeoff between true and false positive rates are often represented by a receiver operation characteristic curve, and the Area Under the ROC Curve (AUC) is a measure of the prediction accuracy that takes into account both sensitivity and specificity of the prediction model. A random predictor receives an AUC of 0.5, and a perfect predictor achieves an AUC of 1. Using 10-fold cross-validation, an AUC is calculated for each fold (one iteration), and the mean and standard deviation of the 10 AUC values are recorded. Calculation and visualization of the ROC curves were performed by the ROCR package [32].

### Support vector machine (SVM)

SVM is a supervised classification algorithm that separates two classes of data based on a set of features [30], [31]. In this study, the set of ChIP enrichment at each MRE is treated as a feature vector of that MRE. Given a set of feature vectors from functional MREs and a set of feature vector from non-functional MREs, the SVM algorithm calculates an optimally-separating hyperplane by maximizing the distance (called margin) between the hyperplane and the nearest points from the two classes. This hyperplane effectively divides the space of feature vectors into two regions, one for each class, with the idea that the larger margin lowers the error of the classifier. To make a prediction, the feature vector corresponding to a MRE from the test set is compared to this hyperplane to determine on which side of the separating boundary this sample is located. We used the SVM implementation in the R package e1071, which is optimized for the radial basis function kernel and uses an Sequential Minimal Optimization-type algorithm [48] using default parameters for training the SVM.

### ChIP–seq data processing

We used the MSL3-TAP ChIP-seq data from Alekseyenko et al [8]. The raw sequence reads were aligned to the *Drosophila melanogaster* genome assembly dm3 using bowtie with default options [49]. We only allowed uniquely mapped reads to be reported. This procedure resulted in 2.8 and 2.4 million mapped reads for ChIP and input DNA samples. The aligned reads were then analyzed with SPP [50] to identify ChIP-enriched regions (FDR threshold of 0.05).

### RNA–seq gene expression data

Gene expression level estimates in S2, BG3 and Kc cells were obtained from the modENCODE project [34]. The expression of each gene is quantified in terms of RPKM (reads per million reads per kilobase). The distribution of gene expression in each cell line was assessed and a cut-off of RPKM = 3 was determined to be a good threshold to separate active vs. inactive genes (Figure S5A). This definition of active vs. inactive genes was used in the construction of meta-gene profiles.

### Construction of meta-gene profiles

We used the gene annotation from FlyBase [39] to define transcription start and end sites (TSS and TES respectively). We only included genes with a minimum length of 2 kb (7,231 of 15,186 genes) to exclude short genes from our analysis. The ChIP enrichment in the 2 kb region centered on the TSS and TES, as well as the ChIP enrichment within the gene body scaled to 1 kb, were calculated and averaged for the active and inactive genes in X and autosomes. The definitions of active vs. inactive genes were defined by RNA-seq data.

### Accession numbers

All ChIP-chip and RNA-seq data are available from modENCODE, and the URL for individual datasets is listed in Table S1. The ChIP-chip and microarray gene expression data pertaining to the Sxl RNAi experiments are accessible from GEO (Accession number: GSE34859).

## Supporting Information

Figure S1 Distribution of functional and non-functional MREs. (A) The number of MREs per megabase (Mb) on each major chromosome arm based on motif detection with different *p*-value thresholds. The number of MREs located on the X chromosome is roughly twice the number of each autosomal chromosome arm. (B) The distribution of motif detection p-values for each of the classes of MREs defined in this study. The motif specificity (as approximated by motif detection *p*-value) of the functional MREs at chromatin entry sites (CESs) is similar to other random MREs on chromosome X and an autosomal arm (chr2L). (C) Histograms showing the distance between consecutive randomly chosen 137 non-functional MREs (left panel) and 137 functional MREs (right panel) along the X chromosome. The red dotted lines indicate the average log2 distance in base pairs between two consecutive MREs. The mean distances of the functional and non-functional MREs are not significantly different (*t*-test), suggesting there is no significant difference in the distribution and clustering of functional and non-functional MREs.
(TIFF)

Figure S2 Average GC content around different classes of MREs. (A) The average plot of GC content around chromatin entry site (CES), autosomal MREs that have H3K36me3 enrichment, and MREs chosen randomly from chromosome 2L. (B) Heat maps showing the distribution of GC content for the MREs shown in panel A. It is particularly striking to observe that even though GC content is generally higher at functional MREs and non-functional autosomal MREs enriched for H3K36me3, there is a strong decrease in GC content only at the center of functional MREs.
(TIFF)

Figure S3 Chromatin features are predictive of functional MREs in both male and female cells. (A–B) AUC of individual and all possible combinations of chromatin features for predicting functional MREs based on 10-fold cross-validation. For each feature or feature set, an SVM model was trained on the chromatin ChIP-chip data of Kc cells, and the prediction performance was tested on data from S2 and BG3 cells. H3K36me3 and JIL-1 are the best individual features, and the best SVM prediction models use a combination of 4 or 5 features. The AUC of the best individual, best combination, or all features are show in brackets. (C) Correlation between every pair of features using the Kc cells data. (D) ROC curves showing the ability of various chromatin features to discriminate functional MREs from non-functional MREs based on 10-fold cross-validation, where SVM was trained on Kc cells, and the performance was assessed on data from BG3 cells. (E) The trade-off between retention of CES and elimination of non-functional autosomal MREs when using SVM for genome-wide prediction of functional MREs. After we train an SVM, we can use different thresholds (distance to hyperplane) to determine whether an MRE is functional or not. The default threshold is 0 (i.e., the best binary classification is achieved using the SVM-determined hyperplane). (F) The trade-off between true positive rate (defined as proportion of CES retained) and false positive rate (defined as proportion of autosomal MRE retained) can be visualized as an ROC curve. The AUC is about 0.84.
(TIFF)

Figure S4 Decreased nucleosome occupancy upon MSL binding on X chromosome. (A) The average nucleosome occupancy in functional and non-functional MREs in male S2 cells. There is a strong decrease of nucleosome occupancy in functional MREs on the X chromosome. (B) The average nucleosome occupancy in functional and non-functional MREs in female Kc cells. There is a less pronounced difference in nucleosome occupancy among different classes of MREs. Collectively, these observations suggest that decrease in nucleosome occupancy is mainly a result of MSL binding on the X chromosome.
(TIFF)

Figure S5 Estimating gene expression activity in S2, BG3 and Kc cells using RNA-seq, and additional meta-gene profiles. (A) Distribution of normalized read counts (RPKM) in S2, BG3 and Kc cell lines. A bimodal distribution of gene expression is apparent. A threshold of log2(RPKM+1) = 2 (ie, RPKM = 3) was chosen as a threshold to distinguish active from inactive genes (red dotted lines). (B) Meta-gene profiles of JIL-1 and H1 in active and inactive genes of BG3 cells. (C) Meta-gene profiles of H4K16ac in active and inactive genes of S2 and Kc cells.
(TIFF)

Figure S6 Chromosome-wide enrichment of different histone marks. There is generally no significant difference between the enrichment distribution on chromosome X and the autosomes in female Kc cells. However, there is enrichment of H2B-ubiq and H3K36me3 and depletion of H1 and H4 in the male S2 and BG3 cell lines, suggesting X chromosome specific enrichment of certain active histone marks in male cells upon dosage compensation. See also Table S2.
(TIFF)

Table S1 URL of the modENCODE datasets used in this study.
(DOC)

Table S2 The number and proportion of non-functional MREs eliminated from each chromosome using the best combination of features, best individual features only (H3K36me3 or JIL-1), or GC content only. Chromatin features can eliminate over 85% of the non-functional MREs on autosomes, and over 75% of non-functional MREs on X. The high proportion of false positives on the X chromosome indicates that there are likely more true MSL binding sites than the set of sites we used in this study.
(DOC)

Table S3 Chromosome-wide enrichment of many active chromatin marks on the male X chromosome compared to autosomes. The mode of the ChIP enrichment density profile is calculated for each major chromosome arm in S2, BG3 and Kc cells. The mode of the X chromosome enrichment is compared to the average of the modes of autosome enrichment in each sample. We observe that the mode of enrichment density is similar in all profiles in Kc cells. In contrast, there is enrichment in many active chromatin marks on the male X chromosome compared to autosomes, as well as depletion of core and linker histones on the male X.
(DOC)

## References

[1] Amrein H, Axel R (1997). Genes expressed in neurons of adult male Drosophila.. Cell.

[2] Lucchesi JC (2009). The structure-function link of compensated chromatin in Drosophila.. Cur Opin Genet Dev.

[3] Straub T, Becker PB (2011). Transcription modulation chromosome-wide: universal features and principles of dosage compensation in worms and flies.. Curr Opin Genet Dev.

[4] Georgiev P, Chlamydas S, Akhtar A (2011). Drosophila dosage compensation: Males are from Mars, females are from Venus.. Fly.

[5] Gelbart ME, Kuroda MI (2009). Drosophila dosage compensation: A complex voyage to the X chromosome.. Development.

[6] Ivaldi MS, Karam CS, Corces VG (2007). Phosphorylation of histone H3 at Ser10 facilitates RNA polymerase II release from promoter-proximal pausing in Drosophila.. Genes Dev.

[7] Kelley RL, Meller VH, Gordadze PR, Roman G, Davis RL (1999). Epigenetic spreading of the Drosophila dosage compensation complex from roX RNA genes into flanking chromatin.. Cell.

[8] Alekseyenko AA, Peng S, Larschan E, Gorchakov AA, Lee OK (2008). A sequence motif within chromatin entry sites directs MSL establishment on the Drosophila X chromosome.. Cell.

[9] Lyman LM, Copps K, Rastelli L, Kelley RL, Kuroda MI (1997). Drosophila male-specific lethal-2 protein: structure/function analysis and dependence on MSL-1 for chromosome association.. Genetics.

[10] Straub T, Grimaud C, Gilfillan GD, Mitterweger A, Becker PB (2008). The chromosomal high-affinity binding sites for the Drosophila dosage compensation complex.. PLoS Genet.

[11] Alekseyenko AA, Larschan E, Lai WR, Park PJ, Kuroda MI (2006). High-resolution ChIP-chip analysis reveals that the Drosophila MSL complex selectively identifies active genes on the male X chromosome.. Genes Dev.

[12] Larschan E, Alekseyenko AA, Gortchakov AA, Peng S, Li B (2007). MSL complex is attracted to genes marked by H3K36 trimethylation using a sequence-independent mechanism.. Mol Cell.

[13] Kind J, Akhtar A (2007). Cotranscriptional recruitment of the dosage compensation complex to X-linked target genes.. Genes Dev.

[14] Bell O, Conrad T, Kind J, Wirbelauer C, Akhtar A (2008). Transcription-coupled methylation of histone H3 at lysine 36 regulates dosage compensation by enhancing recruitment of the MSL complex in Drosophila melanogaster.. Mol Cell Biol.

[15] Gorchakov AA, Alekseyenko AA, Kharchenko P, Park PJ, Kuroda MI (2009). Long-range spreading of dosage compensation in Drosophila captures transcribed autosomal genes inserted on X.. Genes Dev.

[16] Carroll JS, Liu XS, Brodsky AS, Li W, Meyer CA (2005). Chromosome-wide mapping of estrogen receptor binding reveals long-range regulation requiring the forkhead protein FoxA1.. Cell.

[17] Sekinger EA, Moqtaderi Z, Struhl K (2005). Intrinsic histone-DNA interactions and low nucleosome density are important for preferential accessibility of promoter regions in yeast.. Mol Cell.

[18] Kharchenko PV, Alekseyenko AA, Schwartz YB, Minoda A, Riddle NC (2011). Comprehensive analysis of the chromatin landscape in Drosophila melanogaster.. Nature.

[19] Granok H, Leibovitch BA, Shaffer CD, Elgin SC (1995). Chromatin. Ga-ga over GAGA factor.. Curr Biol.

[20] Turner BM, Birley AJ, Lavender J (1992). Histone H4 isoforms acetylated at specific lysine residues define individual chromosomes and chromatin domains in Drosophila polytene nuclei.. Cell.

[21] Kind J, Vaquerizas JM, Gebhardt P, Gentzel M, Luscombe NM (2008). Genome-wide analysis reveals MOF as a key regulator of dosage compensation and gene expression in Drosophila.. Cell.

[22] Gelbart ME, Larschan E, Peng S, Park PJ, Kuroda MI (2009). Drosophila MSL complex globally acetylates H4K16 on the male X chromosome for dosage compensation.. Nat Struct Mol Biol.

[23] Kelley RL, Wang J, Bell L, Kuroda MI (1997). Sex lethal controls dosage compensation in Drosophila by a non-splicing mechanism.. Nature.

[24] Gebauer F, Grskovic M, Hentze MW (2003). Drosophila sex-lethal inhibits the stable association of the 40S ribosomal subunit with msl-2 mRNA.. Mol Cell.

[25] Gorman M, Kuroda MI, Baker BS (1993). Regulation of the sex-specific binding of the maleless dosage compensation protein to the male X chromosome in Drosophila.. Cell.

[26] Duncan K, Grskovic M, Strein C, Beckmann K, Niggeweg R (2006). Sex-lethal imparts a sex-specific function to UNR by recruiting it to the msl-2 mRNA 3′ UTR: translational repression for dosage compensation.. Genes Dev.

[27] Zhang Y, Malone JH, Powell SK, Periwal V, Spana E (2010). Expression in aneuploid Drosophila S2 cells.. PLoS Biol.

[28] Stenberg P, Larsson J (2011). Buffering and the evolution of chromosome-wide gene regulation.. Chromosoma.

[29] Guertin MJ, Lis JT (2010). Chromatin landscape dictates HSF binding to target DNA elements.. PLoS Genet.

[30] Cortes C, Vapnik V (1995). Support-Vector Networks.. Mach Learn.

[31] Shawe-Taylor J, Cristianini N (2000).

[32] Sing T, Sander O, Beerenwinkel N, Lengauer T (2005). ROCR: Visualizing classifier performance in R.. Bioinformatics.

[33] Tweedie S, Ashburner M, Falls K, Leyland P, McQuilton P (2009). FlyBase: Enhancing Drosophila Gene Ontology annotations.. Nucleic Acids Res.

[34] Roy S, Ernst J, Kharchenko PV, Kheradpour P, modENCODE Consortium (2010). Identification of functional elements and regulatory circuits by Drosophila modENCODE.. Science.

[35] Henikoff S, Henikoff JG, Sakai A, Loeb GB, Ahmad K (2009). Genome-wide profiling of salt fractions maps physical properties of chromatin.. Genome Res.

[36] Bell O, Schwaiger M, Oakeley EJ, Lienert F, Beisel C (2010). Accessibility of the Drosophila genome discriminates PcG repression, H4K16 acetylation and replication timing.. Nat Struct Mol Biol.

[37] Jin Y, Wang Y, Walker DL, Dong H, Conley C (1999). JIL-1: a novel chromosomal tandem kinase implicated in transcriptional regulation in Drosophila.. Mol Cell.

[38] Wang Y, Zhang W, Jin Y, Johansen J, Johansen KM (2001). The JIL-1 tandem kinase mediates histone H3 phosphorylation and is required for maintenance of chromatin structure in Drosophila.. Cell.

[39] Drysdale RA, Crosby MA (2005). FlyBase: Genes and gene models.. Nucleic Acids Res.

[40] Regnard C, Straub T, Mitterweger A, Dahlsveen IK, Fabian V (2011). Global analysis of the relationship between JIL-1 kinase and transcription.. PLoS Genet.

[41] Corona DF, Siriaco G, Armstrong JA, Snarskaya N, McClymont SA (2007). ISWI regulates higher-order chromatin structure and histone H1 assembly in vivo.. PLoS Biol.

[42] Vaquerizas JM, Suyama R, Kind J, Miura K, Luscombe NM (2010). Nuclear pore proteins nup153 and megator define transcriptionally active regions in the Drosophila genome.. PLoS Genet.

[43] Bachiller D, Sanchez L (1986). Mutations affecting dosage compensation in Drosophila melanogaster: effects in the germline.. Dev Biol.

[44] Belote JM (1983). Male-Specific Lethal Mutations of DROSOPHILA MELANOGASTER. II. Parameters of Gene Action during Male Development.. Genetics.

[45] Gupta V, Parisi M, Sturgill D, Nuttall R, Doctolero M (2006). Global analysis of X-chromosome dosage compensation.. J Biol.

[46] Sural TH, Peng S, Li B, Workman JL, Park PJ (2008). The MSL3 chromodomain directs a key targeting step for dosage compensation of the Drosophila melanogaster X chromosome.. Nat Struct Mol Biol.

[47] Hamada FN, Park PJ, Gordadze PR, Kuroda MI (2005). Global regulation of X chromosomal genes by the MSL complex in Drosophila melanogaster.. Genes Dev.

[48] Fan R-E, Chen P-H, Lin C-J (2005). Working set selection using second order information for training SVM.. J Mach Learn Res.

[49] Langmead B, Trapnell C, Pop M, Salzberg SL (2009). Ultrafast and memory-efficient alignment of short DNA sequences to the human genome.. Genome Biol.

[50] Kharchenko PV, Tolstorukov MY, Park PJ (2008). Design and analysis of ChIP-seq experiments for DNA-binding proteins.. Nature Biotechnol.

