# Correction: Sequence-Specific Targeting of Dosage Compensation in *Drosophila* Favors an Active Chromatin Context

**DOI:** 10.1371/annotation/39c76721-c8e1-4d42-ae6b-33063ce207d2

**Published:** 2014-01-20

**Authors:** Artyom A. Alekseyenko, Joshua W. K. Ho, Shouyong Peng, Marnie Gelbart, Michael Y. Tolstorukov, Annette Plachetka, Peter V. Kharchenko, Youngsook L. Jung, Andrey A. Gorchakov, Erica Larschan, Tingting Gu, Aki Minoda, Nicole C. Riddle, Yuri B. Schwartz, Sarah C. R. Elgin, Gary H. Karpen, Vincenzo Pirrotta, Mitzi I. Kuroda, Peter J. Park

Supporting information figures were formerly corrupted. This article was republished on the 13th of January to correct this error. No content has been changed

