# Supplementary figures and images for: Sequence-Specific Targeting of Dosage Compensation in Drosophila Favors an Active Chromatin Context
Source: PLoS Genet. 2012 Apr 26;8(4):e1002646. doi: 10.1371/journal.pgen.1002646 (PMC3343056; doi:10.1371/journal.pgen.1002646)

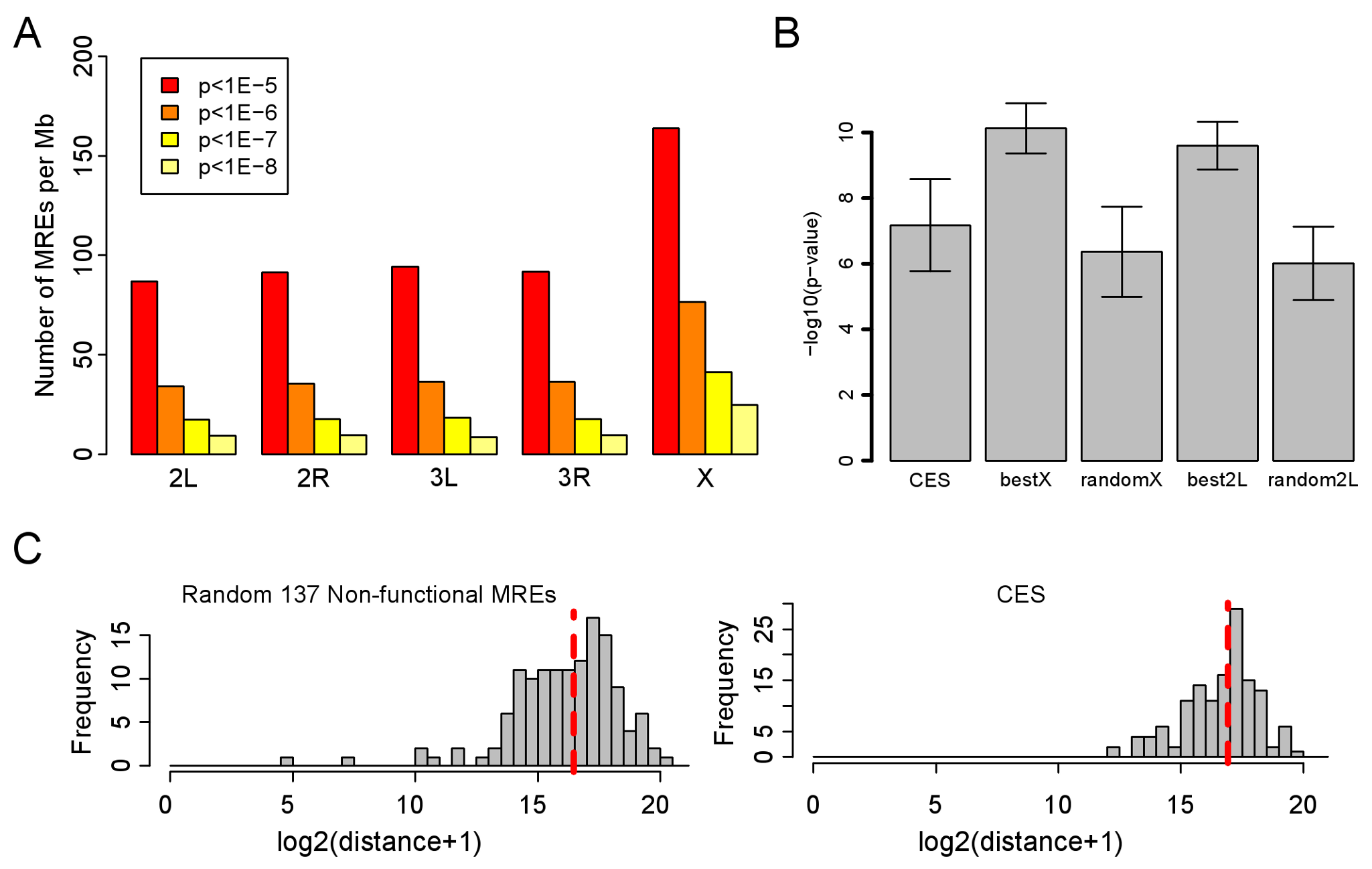

Supplement: Figure S1 — Distribution of functional and non-functional MREs. (A) The number of MREs per megabase (Mb) on each major chromosome arm based on motif detection with different p-value thresholds. The number of MREs located on the X chromosome is roughly twice the number of each autosomal chromosome arm. (B) The distribution of motif detection p-values for each of the classes of MREs defined in this study. The motif specificity (as approximated by motif detection p-value) of the functional MREs at chromatin entry sites (CESs) is similar to other random MREs on chromosome X and an autosomal arm (chr2L). (C) Histograms showing the distance between consecutive randomly chosen 137 non-functional MREs (left panel) and 137 functional MREs (right panel) along the X chromosome. The red dotted lines indicate the average log2 distance in base pairs between two consecutive MREs. The mean distances of the functional and non-functional MREs are not significantly different (t-test), suggesting there is no significant difference in the distribution and clustering of functional and non-functional MREs. (TIFF) [file pgen.1002646.s001.tiff]

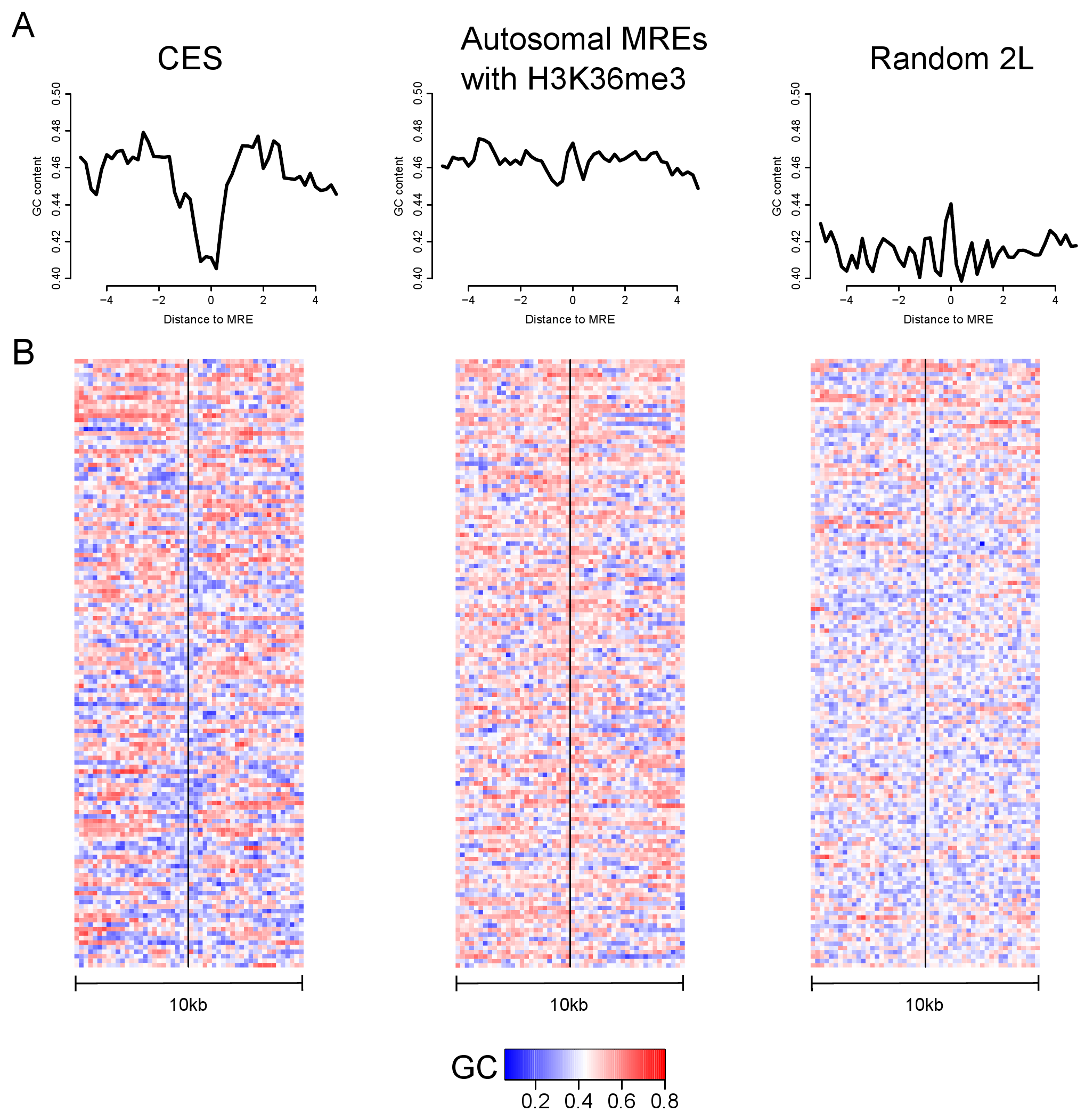

Supplement: Figure S2 — Average GC content around different classes of MREs. (A) The average plot of GC content around chromatin entry site (CES), autosomal MREs that have H3K36me3 enrichment, and MREs chosen randomly from chromosome 2L. (B) Heat maps showing the distribution of GC content for the MREs shown in panel A. It is particularly striking to observe that even though GC content is generally higher at functional MREs and non-functional autosomal MREs enriched for H3K36me3, there is a strong decrease in GC content only at the center of functional MREs. (TIFF) [file pgen.1002646.s002.tiff]

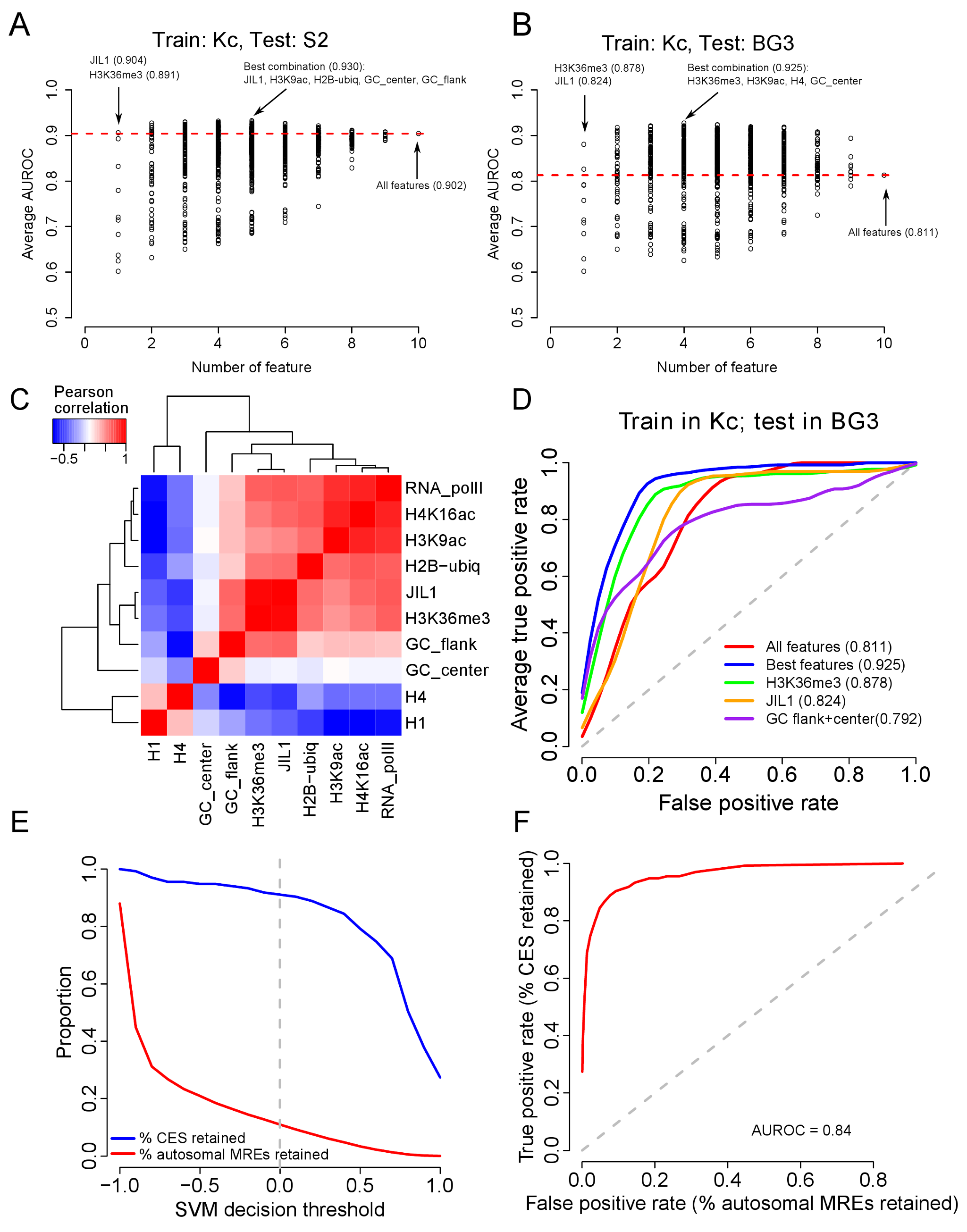

Supplement: Figure S3 — Chromatin features are predictive of functional MREs in both male and female cells. (A–B) AUC of individual and all possible combinations of chromatin features for predicting functional MREs based on 10-fold cross-validation. For each feature or feature set, an SVM model was trained on the chromatin ChIP-chip data of Kc cells, and the prediction performance was tested on data from S2 and BG3 cells. H3K36me3 and JIL-1 are the best individual features, and the best SVM prediction models use a combination of 4 or 5 features. The AUC of the best individual, best combination, or all features are show in brackets. (C) Correlation between every pair of features using the Kc cells data. (D) ROC curves showing the ability of various chromatin features to discriminate functional MREs from non-functional MREs based on 10-fold cross-validation, where SVM was trained on Kc cells, and the performance was assessed on data from BG3 cells. (E) The trade-off between retention of CES and elimination of non-functional autosomal MREs when using SVM for genome-wide prediction of functional MREs. After we train an SVM, we can use different thresholds (distance to hyperplane) to determine whether an MRE is functional or not. The default threshold is 0 (i.e., the best binary classification is achieved using the SVM-determined hyperplane). (F) The trade-off between true positive rate (defined as proportion of CES retained) and false positive rate (defined as proportion of autosomal MRE retained) can be visualized as an ROC curve. The AUC is about 0.84. (TIFF) [file pgen.1002646.s003.tiff]

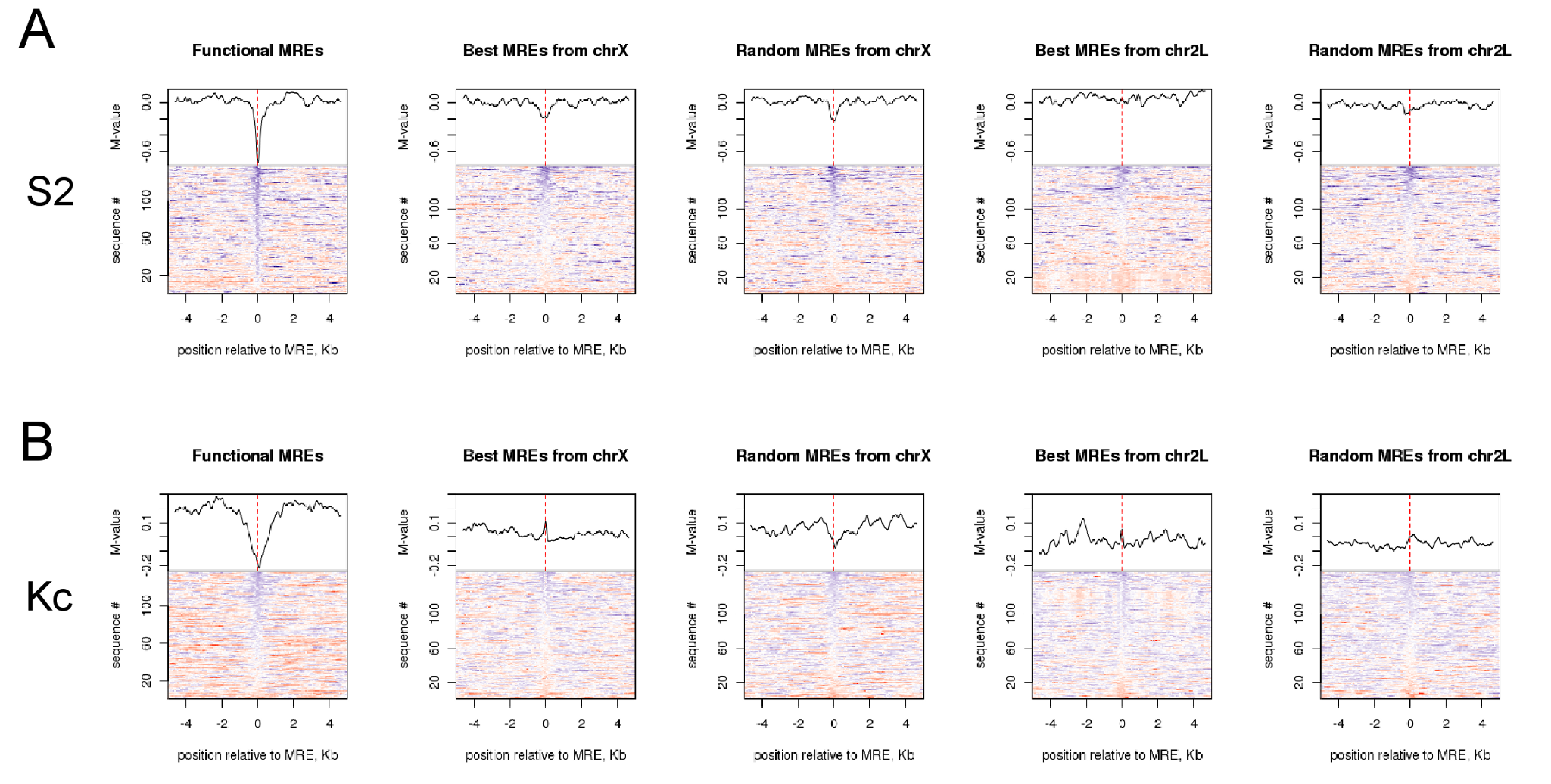

Supplement: Figure S4 — Decreased nucleosome occupancy upon MSL binding on X chromosome. (A) The average nucleosome occupancy in functional and non-functional MREs in male S2 cells. There is a strong decrease of nucleosome occupancy in functional MREs on the X chromosome. (B) The average nucleosome occupancy in functional and non-functional MREs in female Kc cells. There is a less pronounced difference in nucleosome occupancy among different classes of MREs. Collectively, these observations suggest that decrease in nucleosome occupancy is mainly a result of MSL binding on the X chromosome. (TIFF) [file pgen.1002646.s004.tiff]

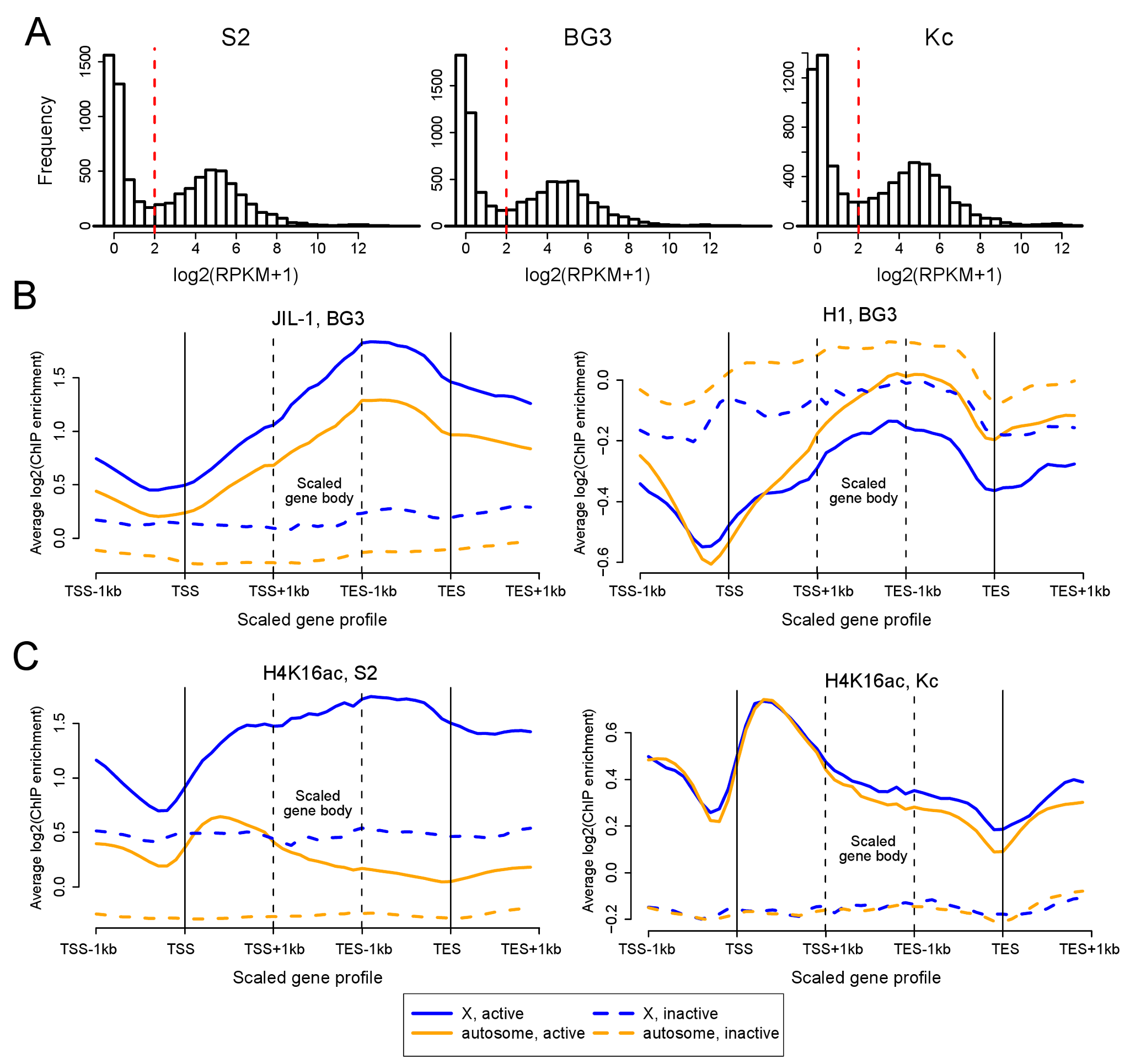

Supplement: Figure S5 — Estimating gene expression activity in S2, BG3 and Kc cells using RNA-seq, and additional meta-gene profiles. (A) Distribution of normalized read counts (RPKM) in S2, BG3 and Kc cell lines. A bimodal distribution of gene expression is apparent. A threshold of log2(RPKM+1) = 2 (ie, RPKM = 3) was chosen as a threshold to distinguish active from inactive genes (red dotted lines). (B) Meta-gene profiles of JIL-1 and H1 in active and inactive genes of BG3 cells. (C) Meta-gene profiles of H4K16ac in active and inactive genes of S2 and Kc cells. (TIFF) [file pgen.1002646.s005.tiff]

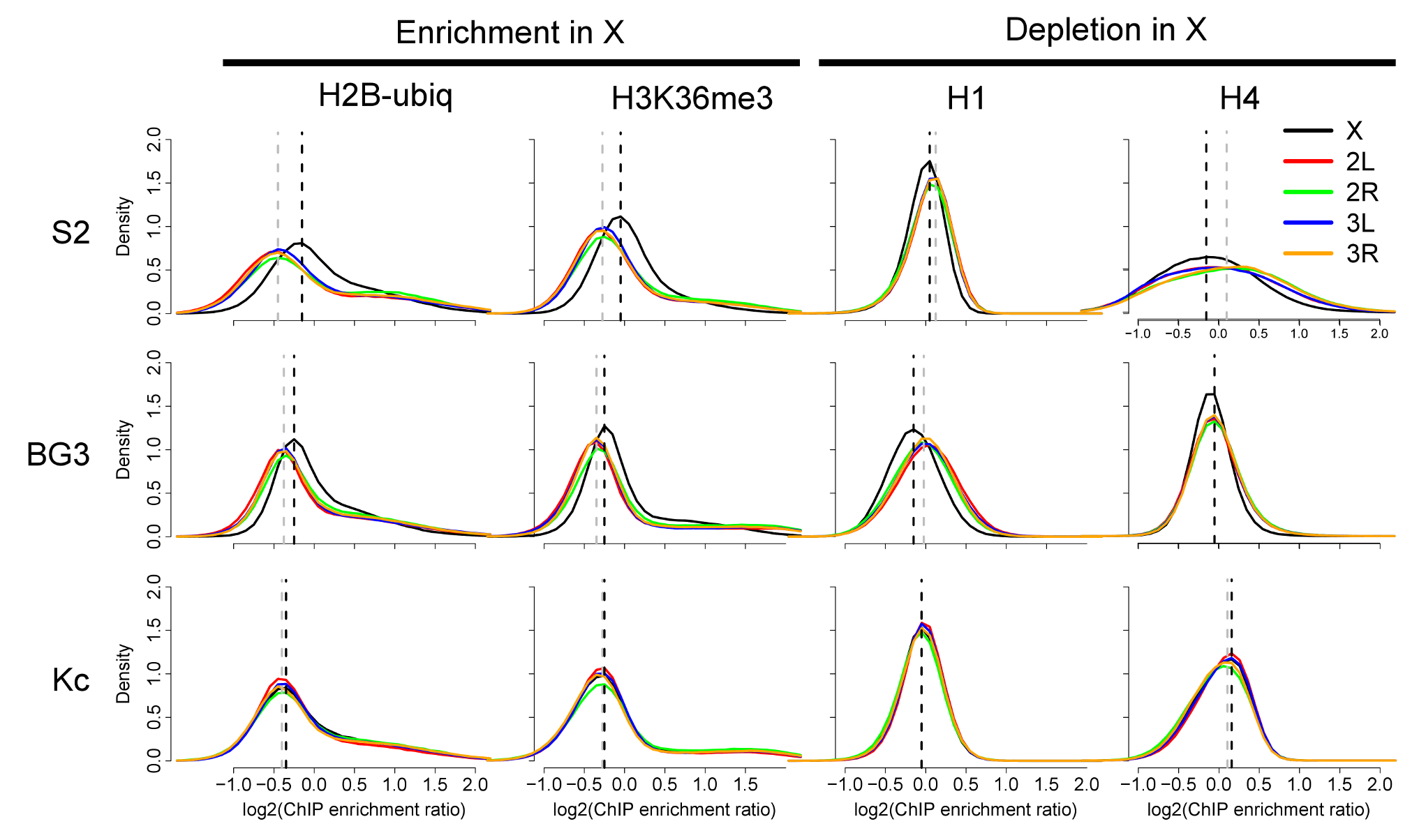

Supplement: Figure S6 — Chromosome-wide enrichment of different histone marks. There is generally no significant difference between the enrichment distribution on chromosome X and the autosomes in female Kc cells. However, there is enrichment of H2B-ubiq and H3K36me3 and depletion of H1 and H4 in the male S2 and BG3 cell lines, suggesting X chromosome specific enrichment of certain active histone marks in male cells upon dosage compensation. See also Table S2. (TIFF) [file pgen.1002646.s006.tiff]
