# Supplementary material for: Sequence-Specific Targeting of Dosage Compensation in Drosophila Favors an Active Chromatin Context
Source: PLoS Genet. 2012 Apr 26;8(4):e1002646. doi: 10.1371/journal.pgen.1002646 (PMC3343056; doi:10.1371/journal.pgen.1002646)
Supplement: Table S1 — URL of the modENCODE datasets used in this study. (DOC) [file pgen.1002646.s007.doc]

| **Data** | **URL** |
| --- | --- |
| **S2** | |
| GAGA factor | http://intermine.modencode.org/release-24/report.do?id=1696000171 |
| H4K16ac | http://intermine.modencode.org/release-24/report.do?id=1696000517 |
| JIL1 | http://intermine.modencode.org/release-24/report.do?id=1696000708 |
| H2B-ubiq | http://intermine.modencode.org/release-24/report.do?id=1696000179 |
| RNA PolII | http://intermine.modencode.org/release-24/report.do?id=1696000602 |
| Chromator | http://intermine.modencode.org/release-24/report.do?id=1696000152 |
| H3K36me3 | http://intermine.modencode.org/release-24/report.do?id=1696000399 |
| H3K9ac | http://intermine.modencode.org/release-24/report.do?id=1696000082 |
| H1 | http://intermine.modencode.org/release-24/report.do?id=1696000604 |
| H3K23ac | http://intermine.modencode.org/release-24/report.do?id=1696000191 |
| MSL-1 | http://intermine.modencode.org/release-24/report.do?id=1696000581 |
| RNA-seq | http://intermine.modencode.org/release-25/report.do?id=112000283 |
| 80_mM_Salt_Extracted_Chromatin | http://intermine.modencode.org/release-26/report.do?id=60000105 |
| 80-150_mM_Salt_Extracted_Chromatin | http://intermine.modencode.org/release-26/report.do?id=60000135 |
| 150-600_mM_Salt_Extracted_Chromatin | http://intermine.modencode.org/release-26/report.do?id=60000163 |
| **BG3** | |
| GAGA factor | http://intermine.modencode.org/release-24/report.do?id=1696000038 |
| H4K16ac | http://intermine.modencode.org/release-24/report.do?id=1696000507 |
| JIL1 | http://intermine.modencode.org/release-24/report.do?id=1696000385 |
| H2B-ubiq | http://intermine.modencode.org/release-24/report.do?id=1696000173 |
| RNA PolII | http://intermine.modencode.org/release-24/report.do?id=1696000191 |
| Chromator | http://intermine.modencode.org/release-24/report.do?id=1696000146 |
| H3K36me3 | http://intermine.modencode.org/release-24/report.do?id=1696000331 |
| H3K9ac | http://intermine.modencode.org/release-24/report.do?id=1696000078 |
| H1 | http://intermine.modencode.org/release-24/report.do?id=1696000598 |
| H3K23ac | http://intermine.modencode.org/release-24/report.do?id=1696000187 |
| RNA-seq | http://intermine.modencode.org/release-25/report.do?id=112000310 |
| **Kc** | |
| GAGA factor | http://intermine.modencode.org/release-26/report.do?id=70000912 |
| H4K16ac | http://intermine.modencode.org/release-24/report.do?id=1696000515 |
| JIL1 | http://intermine.modencode.org/release-24/report.do?id=1696000389 |
| H2B-ubiq | http://intermine.modencode.org/release-24/report.do?id=1696000177 |
| RNA PolII | http://intermine.modencode.org/release-24/report.do?id=1696000572 |
| Chromator | http://intermine.modencode.org/release-24/report.do?id=1696000150 |
| H3K36me3 | http://intermine.modencode.org/release-24/report.do?id=1696000366 |
| H3K9ac | http://intermine.modencode.org/release-24/report.do?id=1696000303 |
| H3K23ac | http://intermine.modencode.org/release-24/report.do?id=1696000264 |
| RNA-seq | http://intermine.modencode.org/release-25/report.do?id=112000303 |
| 600_mM_Salt_Extracted_Chromatin | http://intermine.modencode.org/release-26/report.do?id=60000229 |
| 600_mM_Salt_Extracted_Chromatin_Pellet | http://intermine.modencode.org/release-26/report.do?id=60000236 |
