# Supplementary material for: Sequence-Specific Targeting of Dosage Compensation in Drosophila Favors an Active Chromatin Context
Source: PLoS Genet. 2012 Apr 26;8(4):e1002646. doi: 10.1371/journal.pgen.1002646 (PMC3343056; doi:10.1371/journal.pgen.1002646)
Supplement: Table S2 — The number and proportion of non-functional MREs eliminated from each chromosome using the best combination of features, best individual features only (H3K36me3 or JIL-1), or GC content only. Chromatin features can eliminate over 85% of the non-functional MREs on autosomes, and over 75% of non-functional MREs on X. The high proportion of false positives on the X chromosome indicates that there are likely more true MSL binding sites than the set of sites we used in this study. (DOC) [file pgen.1002646.s008.doc]

|  | **Functional MREs** | **Non-functional MREs** | | | | |  |
| --- | --- | --- | --- | --- | --- | --- | --- |
|  | **CES** | **X** | **2L** | **2R** | **3L** | **3R** | **All non-functional** |
| **Number of MRE** | 135 | 3343 | 1802 | 1789 | 2114 | 2439 | 11487 |
| **Number of MREs retained** | | | | | | | |
| **Best set** | 123 | 763 | 204 | 249 | 201 | 241 | 1658 |
| **H3K36me3** | 116 | 747 | 297 | 384 | 342 | 425 | 2195 |
| **JIL-1** | 127 | 965 | 324 | 377 | 307 | 410 | 2383 |
| **GC** | 63 | 348 | 59 | 122 | 97 | 113 | 739 |
| **Proportion of MREs retained** | | | | | | | |
| **Best set** | 0.91 | 0.23 | 0.11 | 0.14 | 0.10 | 0.10 | 0.14 |
| **H3K36me3** | 0.86 | 0.22 | 0.16 | 0.21 | 0.16 | 0.17 | 0.19 |
| **JIL-1** | 0.94 | 0.29 | 0.18 | 0.21 | 0.15 | 0.17 | 0.21 |
| **GC** | 0.47 | 0.10 | 0.03 | 0.07 | 0.05 | 0.05 | 0.06 |
