# Supplementary material for: Sequence-Specific Targeting of Dosage Compensation in Drosophila Favors an Active Chromatin Context
Source: PLoS Genet. 2012 Apr 26;8(4):e1002646. doi: 10.1371/journal.pgen.1002646 (PMC3343056; doi:10.1371/journal.pgen.1002646)
Supplement: Table S3 — Chromosome-wide enrichment of many active chromatin marks on the male X chromosome compared to autosomes. The mode of the ChIP enrichment density profile is calculated for each major chromosome arm in S2, BG3 and Kc cells. The mode of the X chromosome enrichment is compared to the average of the modes of autosome enrichment in each sample. We observe that the mode of enrichment density is similar in all profiles in Kc cells. In contrast, there is enrichment in many active chromatin marks on the male X chromosome compared to autosomes, as well as depletion of core and linker histones on the male X. (DOC) [file pgen.1002646.s009.doc]

| **ChIP** | **Auto** | **X** | **Differences (X-Auto)** | **Cell type** | **Sex** |
| --- | --- | --- | --- | --- | --- |
| **H4K16ac.BG3** | -0.525 | 0.05 | 0.575 | BG3 | M |
| **H4K16ac.S2** | -0.375 | -0.05 | 0.325 | S2 | M |
| **JIL1.BG3** | -0.475 | -0.15 | 0.325 | BG3 | M |
| **H2B-ubiq.S2** | -0.45 | -0.15 | 0.3 | S2 | M |
| **H3K36me3.S2** | -0.275 | -0.05 | 0.225 | S2 | M |
| **H2B-ubiq.BG3** | -0.375 | -0.25 | 0.125 | BG3 | M |
| **JIL1.S2** | -0.25 | -0.15 | 0.1 | S2 | M |
| **RNA pol II.S2** | -0.25 | -0.15 | 0.1 | S2 | M |
| **H3K36me3.BG3** | -0.35 | -0.25 | 0.1 | BG3 | M |
| **H2B-ubiq.Kc** | -0.4 | -0.35 | 0.05 | Kc | F |
| **H4.Kc** | 0.1 | 0.15 | 0.05 | Kc | F |
| **RNA pol II.Kc** | -0.175 | -0.15 | 0.025 | Kc | F |
| **H3K36me3.Kc** | -0.275 | -0.25 | 0.025 | Kc | F |
| **H3K9ac.S2** | -0.05 | -0.05 | 0 | S2 | M |
| **H3.S2** | 0.05 | 0.05 | 0 | S2 | M |
| **H3K9ac.BG3** | -0.05 | -0.05 | 0 | BG3 | M |
| **H4.BG3** | -0.05 | -0.05 | 0 | BG3 | M |
| **H4K16ac.Kc** | -0.25 | -0.25 | 0 | Kc | F |
| **JIL1.Kc** | -0.35 | -0.35 | 0 | Kc | F |
| **H3K9ac.Kc** | 0.05 | 0.05 | 0 | Kc | F |
| **H1.Kc** | -0.05 | -0.05 | 0 | Kc | F |
| **H3.Kc** | 0.05 | 0.05 | 0 | Kc | F |
| **RNA pol II.BG3** | -0.2 | -0.25 | -0.05 | BG3 | M |
| **H1.S2** | 0.125 | 0.05 | -0.075 | S2 | M |
| **H3.BG3** | -0.05 | -0.15 | -0.1 | BG3 | M |
| **H1.BG3** | -0.025 | -0.15 | -0.125 | BG3 | M |
| **H4.S2** | 0.1 | -0.15 | -0.25 | S2 | M |
